# Total gonadotropin dose is not associated with live birth or embryo quality in fresh cycles at equivalent levels of ovarian response: a retrospective cohort study stratified by ovarian response

**DOI:** 10.3389/fendo.2026.1970297

**Published:** 2026-10-07

**Authors:** Keng Feng, Liyan Wang, Haiwen Wei, Feifei Lei, Derong Li, Yudi Luo, Xiang Li, Lingling Zhu, Jiyi Wang

**Affiliations:** 1 Center for Reproductive Medicine, Yulin Maternal and Child Health Hospital, Yulin, Guangxi, China; 2 Procurement Office, Yulin Maternal and Child Health Hospital, Yulin, Guangxi, China

**Keywords:** controlled ovarian stimulation, embryo quality, fresh embryo transfer, gonadotropin dose, live birth, ovarian response

## Abstract

**Introduction:**

Whether the total gonadotropin (Gn) dose used for controlled ovarian stimulation is independently associated with live birth and embryo quality remains controversial, largely because the dose is individualized according to patient prognosis, which generates confounding by indication. We examined this association within homogeneous levels of ovarian response.

**Methods:**

This single-center retrospective cohort study analyzed 3,711 fresh autologous embryo transfer cycles (female age ≤35 years, follicular-phase long protocol, January 2016 to June 2025); cycles with trigger-day progesterone ≥1.5 ng/mL were excluded because fresh transfer was canceled per center policy. Cycles were stratified by oocyte yield into poor (<10, n=1,346), normal (10–15, n=1,582), and high (>15, n=783) response. The exposure was the total Gn dose. The primary outcome was the fresh embryo transfer live birth rate; secondary outcomes included clinical pregnancy, early miscarriage, and morphological embryo-quality indicators. We modeled dose–response relationships with restricted cubic splines (RCS), compared dose quartiles within strata using sequentially adjusted logistic regression, controlled the false discovery rate (FDR) by the Benjamini–Hochberg method, and prespecified five sensitivity analyses.

**Results:**

In the overall cohort, the total Gn dose showed neither a linear (P=0.871) nor a nonlinear (P=0.701) association with live birth. Compared with the lowest dose quartile (Q1), fully adjusted odds ratios for Q2, Q3, and Q4 were 1.18 (95% CI: 0.90–1.55), 1.08 (0.78–1.49), and 1.34 (0.92–1.95), and no stratum showed a significant association. In the normal-response stratum, the highest quartile was associated with reduced live birth in the crude model (OR=0.70, P=0.036), but the association attenuated and disappeared with sequential adjustment (aOR=1.08, P=0.757), which quantitatively illustrates how confounding by indication arises and how it can be corrected. No secondary outcome remained significant after FDR correction (minimum q=0.137), and all sensitivity analyses agreed with the main analysis.

**Conclusions:**

Within the same level of ovarian response, the total Gn dose was not significantly associated with fresh embryo transfer live birth or morphological embryo quality. Within the dose range studied (700–8,025 IU; interquartile range 1,650–2,725 IU), escalating the Gn dose to improve embryo quality or live birth lacks evidential support, and moderate dose increases in patients with reduced ovarian sensitivity showed no signal of harm.

## 1 Introduction

Controlled ovarian stimulation (COS) is the central step of *in vitro* fertilization–embryo transfer (IVF-ET). Exogenous gonadotropins (Gn) maintain follicle-stimulating hormone (FSH) concentrations continuously above the threshold for follicular recruitment, thereby rescuing a cohort of antral follicles that would otherwise undergo atresia and driving their synchronous development (1, 2). The Gn dose directly affects the safety and cost of treatment: it is one of the principal determinants of the risk of ovarian hyperstimulation syndrome (OHSS), and higher doses entail higher medication costs (3). Few clinical decisions in an IVF cycle are as routine yet as consequential as dose selection. Over the past two decades, ovarian reserve assessment centered on anti-Müllerian hormone (AMH) and the antral follicle count (AFC) has moved starting-dose selection from empiricism toward quantification, supported by large randomized trials and international guidelines (4–7).

Large-sample observational evidence on total Gn dose and fresh-cycle outcomes consistently points in a harmful direction. Analyzing 658,519 fresh cycles from the United States SART registry, Baker et al. found that, within every stratum of oocyte yield, live birth rates declined as the total FSH dose increased, a trend that persisted after restriction to good-prognosis patients (8). Two large Chinese studies, by Xu et al. and Shen et al., reported negative associations in the same direction (9, 10), with Xu et al. further suggesting a nonlinear inflection point at approximately 1,410 IU (9). A cohort study of antagonist cycles by Yang et al. likewise linked higher stimulation intensity to adverse outcomes, with effects varying by the level of ovarian response (11). However, this body of evidence shares a common methodological limitation: Gn dose is not randomly assigned but is individually prescribed by clinicians according to patient prognosis, with poorer-prognosis patients receiving higher doses—classic confounding by indication in observational research (12). Moreover, the number of oocytes retrieved is itself nonlinearly associated with fresh-cycle live birth (13, 14) and is closely related to cumulative live birth (15, 16), meaning that the level of ovarian response cannot be ignored in the dose–outcome relationship. Because previous studies have rarely compared doses within the same level of ovarian response, the harmful associations they observed are difficult to distinguish from artifacts of confounding.

Mechanistic evidence is less consistent. Regarding the embryo pathway, animal experiments by Roberts et al. suggested that supraphysiological FSH levels can disrupt chromosome alignment during oocyte meiosis (17), and a randomized trial by Baart et al. showed that mild stimulation reduces the proportion of aneuploid embryos (18); however, in the era of preimplantation genetic testing (PGT), four studies—by Barash et al., Sekhon et al., Irani et al., and Shuai et al.—consistently found no significant association between Gn dose and the embryonic aneuploidy rate (19–22). Regarding the endometrial pathway, Shapiro et al. found that high FSH doses were associated with reduced live birth in fresh cycles but not in subsequent frozen cycles (23), and a meta-analysis by Roque et al. comparing freeze-all with fresh transfer pointed in the same direction (24), together suggesting that ovarian stimulation may impair endometrial receptivity and that any potential dose effect may act on the endometrium rather than on the embryo itself. Meanwhile, randomized trials of dose escalation in predicted normal or poor responders by Jayaprakasan et al. (25) and Van Tilborg et al. (26), a randomized trial of dose reduction in predicted high responders by Oudshoorn et al. (27), and a Cochrane systematic review by Lensen et al. (28) all found that dose adjustment did not significantly affect live birth.

Against this background, the present study aimed to clarify the relationship of total Gn dose with fresh-cycle live birth and embryo quality at equivalent levels of ovarian response, thereby providing a scientific basis for individualized Gn dose decisions.

## 2 Materials and methods

### 2.1 Study design and data sources

This was a single-center retrospective cohort study, reported in accordance with the STROBE statement for observational studies. Cycles of *in vitro* fertilization (IVF)/intracytoplasmic sperm injection (ICSI) performed at the Center for Reproductive Medicine of Yulin Maternal and Child Health Hospital between January 2016 and June 2025 that met the eligibility criteria were included. The study protocol was approved by the institutional ethics committee; given the retrospective design and the de-identification of the data, informed consent was waived. The study was conducted in accordance with the Declaration of Helsinki throughout.

### 2.2 Study population

The inclusion criteria were: (1) female age ≤35 years; (2) ovarian stimulation with the follicular-phase long protocol; (3) an autologous fresh embryo transfer cycle; and (4) complete Gn medication records and pregnancy outcome follow-up. The exclusion criteria were: (1) cycles with multi-dose pituitary down-regulation or ultra-long protocols; (2) pregnancy outcome unascertainable after transfer; (3) severe endometriosis (stage III–IV); (4) recurrent miscarriage (≥2 consecutive spontaneous miscarriages) or repeated implantation failure; (5) known uterine malformations, endometrial lesions, or uterine fibroids distorting the uterine cavity; (6) oocyte donation or PGT cycles; (7) severe concomitant medical or surgical disease (e.g., severe cardiovascular disease, uncontrolled diabetes mellitus, or thyroid disease); and (8) per center policy, cycles in which fresh transfer was canceled in favor of freeze-all because of trigger-day progesterone ≥1.5 ng/mL.

### 2.3 Controlled ovarian stimulation

All cycles used the follicular-phase long protocol. A single intramuscular injection of a long-acting GnRH agonist microsphere formulation (triptorelin acetate microspheres for injection, 3.75 mg) was administered on days 2–5 of the menstrual cycle. Transvaginal ultrasonography and serum hormone assays (E2, FSH, LH) were performed 28–30 days after the injection to assess down-regulation; once the down-regulation criteria were met (all follicles in both ovaries <10 mm, serum E2 <50 pg/mL, LH <5 mIU/mL, and endometrial thickness <5 mm), exogenous Gn was initiated.

### 2.4 Trigger, oocyte retrieval, and insemination

Final oocyte maturation was triggered when at least two leading follicles reached ≥18 mm in diameter or at least three reached ≥17 mm. The trigger agent was either subcutaneous recombinant human chorionic gonadotropin 250 μg or intramuscular urinary human chorionic gonadotropin 2,000–10,000 IU, with the formulation and dose individualized according to the estimated OHSS risk. Oocyte retrieval was performed 36 hours after triggering under transvaginal ultrasound guidance. The insemination method (conventional IVF, ICSI, or rescue ICSI) was determined by semen quality and previous fertilization history. Fertilization was assessed 16–18 hours after insemination, with the appearance of two pronuclei (2PN) defined as normal fertilization.

### 2.5 Embryo culture and grading

Zygotes were cultured in sequential media. On day 3 (D3), cleavage-stage embryos were graded I–IV according to blastomere number, size uniformity, and fragmentation, following the Istanbul consensus on embryo assessment (29); grades I and II were defined as good-quality embryos. In cycles undergoing blastocyst culture, embryos were cultured to day 5–6 and graded with the Gardner scoring system according to blastocyst expansion and the quality of the inner cell mass and trophectoderm; blastocysts scored ≥3BB were defined as good quality (30). One or two embryos (D3 cleavage-stage embryos or D5–6 blastocysts) were transferred in fresh cycles, and the remaining usable embryos were cryopreserved by vitrification.

### 2.6 Luteal support and pregnancy assessment

All fresh transfer cycles received luteal support (vaginal, oral, or intramuscular progesterone preparations) from the day of oocyte retrieval until the pregnancy test day, per center routine; in confirmed pregnancies, support was continued until 8–10 weeks of gestation. Serum β-hCG was measured 14 days after transfer; in positive cases, clinical pregnancy (intrauterine gestational sac) was confirmed by transvaginal ultrasonography 2–3 weeks later. Pregnant cycles were followed until delivery, and gestational age at delivery, the number of live births, and miscarriages were recorded; miscarriage was classified as early (before 12 weeks of gestation) or late (at or beyond 12 weeks).

### 2.7 Exposure definition and ovarian response stratification

The exposure was the total Gn dose (IU), defined as the arithmetic sum of all FSH and HMG preparations administered from Gn initiation to the trigger day (urinary preparations were converted at 75 IU per vial). The cohort was stratified by the number of oocytes retrieved into poor (<10 oocytes), normal (10–15), and high (>15) response strata; the cutoffs were based primarily on international expert consensus for assessing ovarian response (31–33) and large-cohort evidence on the relationship between oocyte yield and live birth (13), in combination with the distribution of oocyte yield in the present cohort. Ovarian sensitivity to gonadotropins was quantified with the ovarian sensitivity index (OSI): OSI = number of oocytes retrieved ÷ total Gn dose × 1,000, i.e., the number of oocytes obtained per 1,000 IU of Gn consumed, reported as a descriptive measure (34).

### 2.8 Outcome measures

The primary outcome was the fresh-cycle live birth rate (= number of cycles with live birth/number of transfer cycles × 100%), with live birth defined as delivery at ≥28 weeks of gestation of at least one live-born infant. The secondary outcomes and their calculation formulas were: (1) clinical pregnancy rate = number of clinical pregnancy cycles/number of transfer cycles × 100%; (2) early miscarriage rate = number of early miscarriage cycles/number of clinical pregnancy cycles × 100%; (3) 2PN fertilization rate, with the denominator defined by insemination method—for conventional IVF, number of normally fertilized (2PN) oocytes/number of inseminated oocytes × 100%, and for ICSI, number of 2PN oocytes/number of injected MII oocytes × 100%; (4) D3 good-quality embryo rate = number of D3 good-quality embryos/number of normally cleaved embryos derived from 2PN × 100%; (5) blastocyst formation rate = number of blastocysts formed/number of embryos placed in blastocyst culture × 100%; and (6) good-quality blastocyst rate = number of good-quality blastocysts/number of blastocysts formed × 100%. Fertilization and embryology laboratory indicators were calculated according to the definitions of the Vienna consensus on performance indicators for ART laboratories (35).

### 2.9 Statistical analysis

Normally distributed continuous variables are described as mean ± standard deviation and were compared between groups by analysis of variance; skewed continuous variables are described as median (interquartile range) and were compared by the Kruskal–Wallis test; categorical variables are described as frequency (percentage) and were compared by the χ² test or Fisher’s exact test.

The primary analysis used restricted cubic splines (RCS) to model the dose–response relationship between total Gn dose and fresh-cycle live birth. Four knots were placed, per Harrell’s recommendation, at the 5th, 35th, 65th, and 95th percentiles of the total Gn dose distribution; the overall model used the full-cohort distribution, and the stratified models used the within-stratum distributions. The reference dose was set to the median of the corresponding population, and Wald tests for the overall and nonlinear effects are reported.

Within each response stratum, cycles were grouped into quartiles of total Gn dose (Q1 as reference) and analyzed with sequentially adjusted logistic regression: a crude model (univariable), model I (adjusted for female age and BMI), and model II (fully adjusted for 12 covariates: female age, BMI, duration of infertility, primary infertility, baseline AMH, total antral follicle count, number of oocytes retrieved, trigger-day endometrial thickness, number of embryos transferred, use of urinary Gn, D5 blastocyst transfer, and ICSI). Among the secondary outcomes, the 2PN fertilization rate, D3 good-quality embryo rate, and good-quality blastocyst rate were analyzed with binomial generalized linear models, and the clinical pregnancy rate and early miscarriage rate with logistic regression.

Five prespecified sensitivity analyses were repeated independently within each response stratum: (1) restriction to first oocyte retrieval cycles, to exclude the influence of repeat cycles; (2) E-value analysis, quantifying the minimum strength of unmeasured confounding required to explain away the observed point estimates (36); (3) propensity score overlap weighting—taking the Q4 vs Q1 comparison as an example, propensity scores (PS) for membership in Q4 were estimated with a logistic model containing all model II covariates, with weights of 1 − PS for the Q4 group and PS for the Q1 group, tilting the target population toward the clinical equipoise population in which the two groups’ characteristics overlap; covariate balance after weighting was judged by standardized mean differences <0.1 (37); (4) exclusion of cycles with BMI ≥28 kg/m², to examine obesity-related residual confounding; and (5) multiple imputation by chained equations (MICE; five imputed datasets including all outcomes, exposures, and covariates), with estimates combined by Rubin’s rules. All tests were two-sided, and P < 0.05 was considered statistically significant. Analyses were performed in R version 4.3.1 (R Foundation for Statistical Computing, Vienna, Austria).

### 2.10 Sample size and statistical power

Given the retrospective cohort design, no *a priori* sample size calculation was performed. Taking the Q4 vs Q1 comparison of live birth within each response stratum as the benchmark, at a two-sided α=0.05 and 80% power the poor-, normal-, and high-response strata could detect absolute differences of approximately 10.8, 9.6, and 13.4 percentage points, respectively. Smaller effects cannot be excluded; confidence intervals are therefore reported for all primary comparisons.

## 3 Results

### 3.1 Cohort selection and analytic framework

**Figure 1** shows the overall workflow of the study design and statistical analyses. Of the 4,109 cycles assessed, 17 (0.4%) were excluded for multi-dose down-regulation or ultra-long protocols. Of the remaining 4,092 cycles, 317 (7.7%) had fresh transfer cancelled with freeze-all per center policy because of trigger-day progesterone ≥1.5 ng/mL, and 64 (1.7%) were excluded because the pregnancy outcome could not be confirmed. The final cohort comprised 3,711 cycles: 1,346 (36.3%) in the poor-response stratum, 1,582 (42.6%) in the normal-response stratum, and 783 (21.1%) in the high-response stratum. In the full-cohort analysis, we fitted the dose–response relationship between total Gn dose and fresh-cycle live birth using four-knot restricted cubic splines (RCS) and performed sequentially adjusted analyses across the full-cohort dose quartiles. The stratified analyses were conducted independently within each response stratum and comprised within-stratum RCS (knots at the 5th/35th/65th/95th percentiles of the within-stratum total Gn dose), three-model sequentially adjusted logistic regression with Q1 as reference, secondary outcome analyses, and all five prespecified sensitivity analyses. The poor-response stratum was further subdivided into subgroups of ≤4 and 5–9 oocytes retrieved for subgroup analysis.

**Figure 1:**
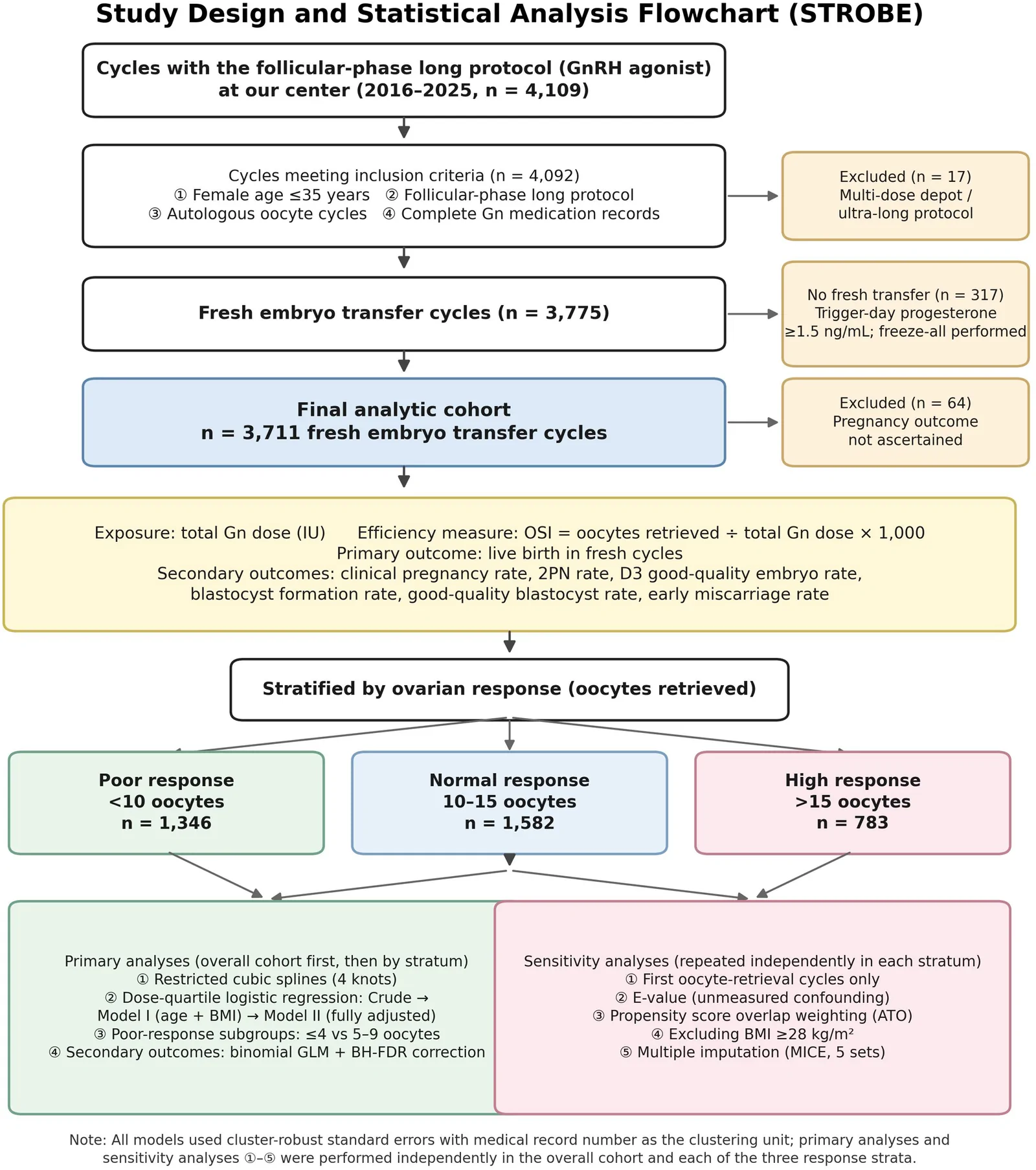
Study design and statistical analysis flowchart (STROBE). Of 4,109 assessed cycles, 17 were excluded because of multi-dose down-regulation or an ultra-long protocol, and 317 did not undergo fresh transfer because trigger-day progesterone was ≥1.5 ng/mL and freeze-all was performed. After exclusion of 64 cycles with unascertained pregnancy outcomes, 3,711 cycles were analyzed and stratified by oocyte yield into poor (<10; n=1,346), normal (10–15; n=1,582), and high (>15; n=783) response strata.

### 3.2 Baseline characteristics

**Table 1** lists the principal baseline characteristics by full-cohort quartiles of total Gn dose; the complete set of 44 characteristics is presented in **Supplementary Table 1**. Across ascending dose quartiles, characteristics indicating poorer prognosis showed a consistent gradient: female age, duration of infertility, and the proportion of secondary infertility all increased, while ovarian reserve progressively declined. History of live birth and history of miscarriage also differed significantly between groups (P<0.001 and P = 0.037, respectively). Regarding medications (**Supplementary Table 1**), the Gn starting dose, duration of stimulation, and HMG dose all increased from Q1 to Q4 (all P for trend <0.001). Regarding medication origin, the proportion of cycles using urinary Gn rose from 47.7% to 94.1%, cycles using exclusively domestic products rose from 37.2% to 71.0%, cycles using exclusively imported products fell from 33.6% to 1.7%, and the proportion of mixed use remained stable (P = 0.107). Regarding ovarian response, trigger-day E2, the number of oocytes retrieved, and the number of mature oocytes declined with increasing dose, and the median OSI fell from 9.1 to 2.8.

**Table 1:** Condensed baseline characteristics of the overall cohort by total Gn dose quartile.

Variable	Q1 (n = 956)	Q2 (n = 911)	Q3 (n = 964)	Q4 (n = 880)	P value
Female age, years	29.7 ± 3.3	30.2 ± 3.2	30.6 ± 3.2	31.3 ± 3.0	<0.001
Body mass index, kg/m²	21.1 ± 2.6	21.9 ± 3.0	22.5 ± 3.2	23.1 ± 3.7	<0.001
Duration of infertility, years	3.0 (2.0–5.0)	3.0 (2.0–5.0)	3.0 (2.0–5.0)	4.0 (2.0–6.0)	<0.001
Primary infertility	459 (48.0)	415 (45.6)	378 (39.2)	369 (41.9)	—
Basal AMH, ng/mL	5.0 (3.7–6.7)	3.6 (2.8–5.0)	3.0 (2.2–4.2)	2.4 (1.7–3.4)	<0.001
Total antral follicle count, n	22.0 (16.8–25.0)	18.0 (14.0–23.0)	16.0 (12.0–20.0)	13.0 (10.0–18.0)	<0.001
Basal FSH, IU/L	5.2 (4.5–6.0)	5.5 (4.8–6.5)	5.7 (4.9–6.5)	6.0 (5.1–6.9)	<0.001
Gn starting dose, IU	150.0 (112.5–150.0)	150.0 (150.0–200.0)	225.0 (150.0–225.0)	225.0 (225.0–300.0)	<0.001
Total Gn duration, days	10.1 ± 1.4	10.8 ± 1.6	11.3 ± 1.6	12.8 ± 2.4	<0.001
Total Gn dose, IU	1425.0 (1246.9–1550.0)	1987.5 (1800.0–2062.5)	2475.0 (2325.0–2600.0)	3225.0 (2925.0–3600.0)	<0.001
Urinary-source Gn use, n (%)	456 (47.7)	599 (65.8)	744 (77.2)	828 (94.1)	<0.001
Progesterone on trigger day, ng/mL	0.5 (0.3–0.7)	0.6 (0.4–0.8)	0.6 (0.4–0.8)	0.6 (0.4–0.8)	<0.001
Endometrial thickness on trigger day, mm	10.5 (9.2–11.9)	10.7 (9.3–12.0)	10.6 (9.4–12.0)	10.8 (9.3–12.0)	0.310
Oocytes retrieved, n	12.7 ± 4.6	11.7 ± 4.6	11.6 ± 4.6	10.0 ± 4.5	<0.001
OSI	9.1 (6.7–11.8)	5.9 (4.3–7.6)	4.7 (3.3–6.0)	2.8 (2.0–3.9)	<0.001
ICSI	234 (24.5)	235 (25.8)	253 (26.2)	214 (24.3)	—
Two embryos transferred	634 (66.3)	547 (60.0)	611 (63.4)	578 (65.7)	—
D5 blastocyst transfer	246 (25.7)	199 (21.8)	213 (22.1)	145 (16.5)	—

Q1–Q4 are whole-cohort quartiles of total Gn dose (Q1: 700–1,650 IU; Q2: 1,675–2,212.5 IU; Q3: 2,225–2,700 IU; Q4: 2,725–8,025 IU). Live-birth history, miscarriage history, total HMG dose, medication source, and additional laboratory variables are reported in **Supplementary Table 1**. AMH, anti-Müllerian hormone; BMI, body mass index; FSH, follicle-stimulating hormone; Gn, gonadotropin; ICSI, intracytoplasmic sperm injection; OSI, ovarian sensitivity index.

### 3.3 Characteristics and outcomes across the three response strata

As shown in **Table 2**, the three response strata effectively discriminated levels of ovarian response: the numbers of oocytes retrieved were 6.7 ± 2.0, 12.3 ± 1.6, and 18.3 ± 2.2, respectively, and the median OSI approximately doubled from stratum to stratum. Regarding Gn use, the poor-response stratum had the highest total and starting Gn doses and the high-response stratum the lowest, while the duration of Gn stimulation was similar across strata. Ovarian reserve indicators increased stepwise across strata: median AMH was 2.8, 3.6, and 4.3 ng/mL, and the total antral follicle count was 15, 18, and 19, respectively. Female age was highest in the poor-response stratum (P<0.001), whereas BMI did not differ significantly across strata (P = 0.673).

**Table 2:** Characteristics and outcomes of the three ovarian response strata.

Characteristic	Poor response (<10 oocytes, n = 1,346)	Normal response (10–15 oocytes, n = 1,582)	High response (>15 oocytes, n = 783)	P value
Female age, years	30.7 ± 3.2	30.5 ± 3.2	29.9 ± 3.4	<0.001
Body mass index, kg/m²	22.2 ± 3.2	22.1 ± 3.2	22.0 ± 3.3	0.673
Basal AMH, ng/mL	2.8 (1.8–4.2)	3.6 (2.5–5.0)	4.3 (3.1–5.7)	<0.001
Total antral follicle count, n	15.0 (11.0–21.0)	18.0 (14.0–23.0)	19.0 (15.0–24.0)	<0.001
Gn starting dose, IU	200.0 (150.0–225.0)	150.0 (150.0–225.0)	150.0 (150.0–225.0)	<0.001
Total Gn duration, days	11.4 ± 2.2	11.2 ± 1.9	11.1 ± 1.8	0.005
Total Gn dose, IU	2375.0 (1856.2–3000.0)	2175.0 (1650.0–2625.0)	2000.0 (1500.0–2412.5)	<0.001
Oocytes retrieved, n	6.7 ± 2.0	12.3 ± 1.6	18.3 ± 2.2	<0.001
OSI	2.8 (2.1–4.0)	5.7 (4.5–7.4)	9.4 (7.4–12.0)	<0.001
ICSI	314 (23.3)	413 (26.1)	209 (26.7)	—
Two embryos transferred	910 (67.6)	1038 (65.6)	422 (53.9)	—
D5 blastocyst transfer	105 (7.8)	319 (20.2)	379 (48.4)	—
Clinical pregnancy rate, n (%)	759 (56.4)	933 (59.0)	479 (61.2)	0.085
Early miscarriage rate, n (%)*	79 (10.4)	71 (7.6)	57 (11.9)	0.020
Live birth in fresh cycles, n (%)	635 (47.2)	804 (50.8)	362 (46.2)	0.051
2PN fertilization rate, %	66.4	66.6	64.8	—
D3 good-quality embryo rate, %	53.5	52.4	50.3	—
Blastocyst formation rate, %	69.7	70.8	72.4	—
Good-quality blastocyst rate, %	26.5	26.8	27.6	—

*Early miscarriage is calculated among clinical pregnancy cycles. Laboratory indicators are pooled cycle-level rates. D3, day 3; D5, day 5; other abbreviations as in **Table 1**.

Live birth and clinical pregnancy rates in fresh embryo transfer cycles were similar across the three response strata. Early miscarriage rates differed significantly among strata (10.4%, 7.6%, and 11.9%; P = 0.020), being lowest in the normal-response stratum in a shallow U-shaped distribution; however, this comparison was unadjusted, the P value was only marginally significant, event counts were limited in each stratum, and multiplicity was a concern, so we regard it as an exploratory finding. Embryology laboratory indicators were broadly consistent across strata.

### 3.4 Full-cohort analysis

#### 3.4.1 Dose–response relationship between total Gn dose and live birth in the full cohort (RCS)

The RCS analysis (**Figure 2A**) showed that, after adjustment for confounders, the dose–response curve relating total Gn dose to the fresh embryo transfer live birth rate was approximately flat over the principal data range of approximately 1,000–4,500 IU. Live birth varied by less than 2 percentage points across the entire range, the 95% confidence band overlapped throughout, and there was no statistically significant ascending or descending segment. The overall dose effect was χ²(3)=0.71 (P = 0.871), and the test for nonlinearity was P = 0.701. The curve showed neither a linear trend nor a statistically significant threshold or inflection point. After adjustment for confounders, no significant dose–response association between total Gn dose and fresh embryo transfer live birth was observed in the full cohort.

**Figure 2:**
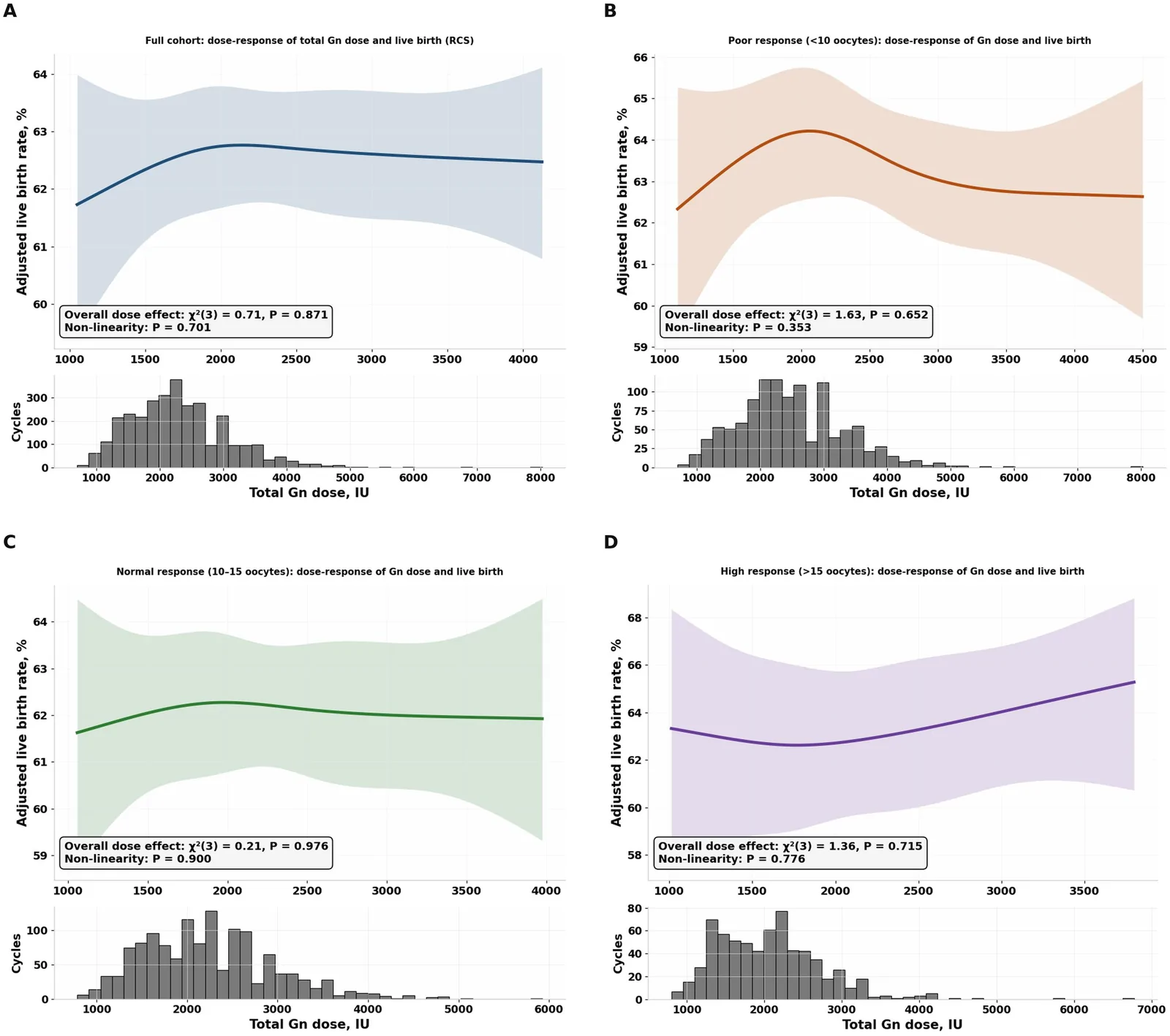
Conditional adjusted dose–response relationships between total Gn dose and live birth in fresh cycles in the overall cohort **(A)**, poor-response stratum **(B)**, normal-response stratum **(C)**, and high-response stratum **(D)**. Curves are conditional predicted probabilities of live birth at the reference covariate profile from fully adjusted restricted cubic spline models fitted in complete cases (overall n=3,129; poor n=1,133; normal n=1,327; high n=669); they are not observed or marginally standardized rates, so their absolute level is higher than the observed live birth rates. Shaded areas are 95% confidence bands. Histograms show the dose distributions.

#### 3.4.2 Total Gn dose quartiles and live birth in the full cohort

**Figure 3A**, **Table 3** show the results with Q1 (700–1,650 IU; live birth rate 50.6%) as reference. Live birth rates in Q2–Q4 were 52.4%, 50.7%, and 49.8%, respectively—a maximum between-group difference of only 2.6 percentage points, with no monotonic trend of increasing dose. The odds ratios for Q2–Q4 vs Q1 were highly stable across the three sequentially adjusted models, and no comparison reached statistical significance. In all three models, full-cohort quartiles of total Gn dose were not significantly associated with fresh-cycle live birth.

**Figure 3:**
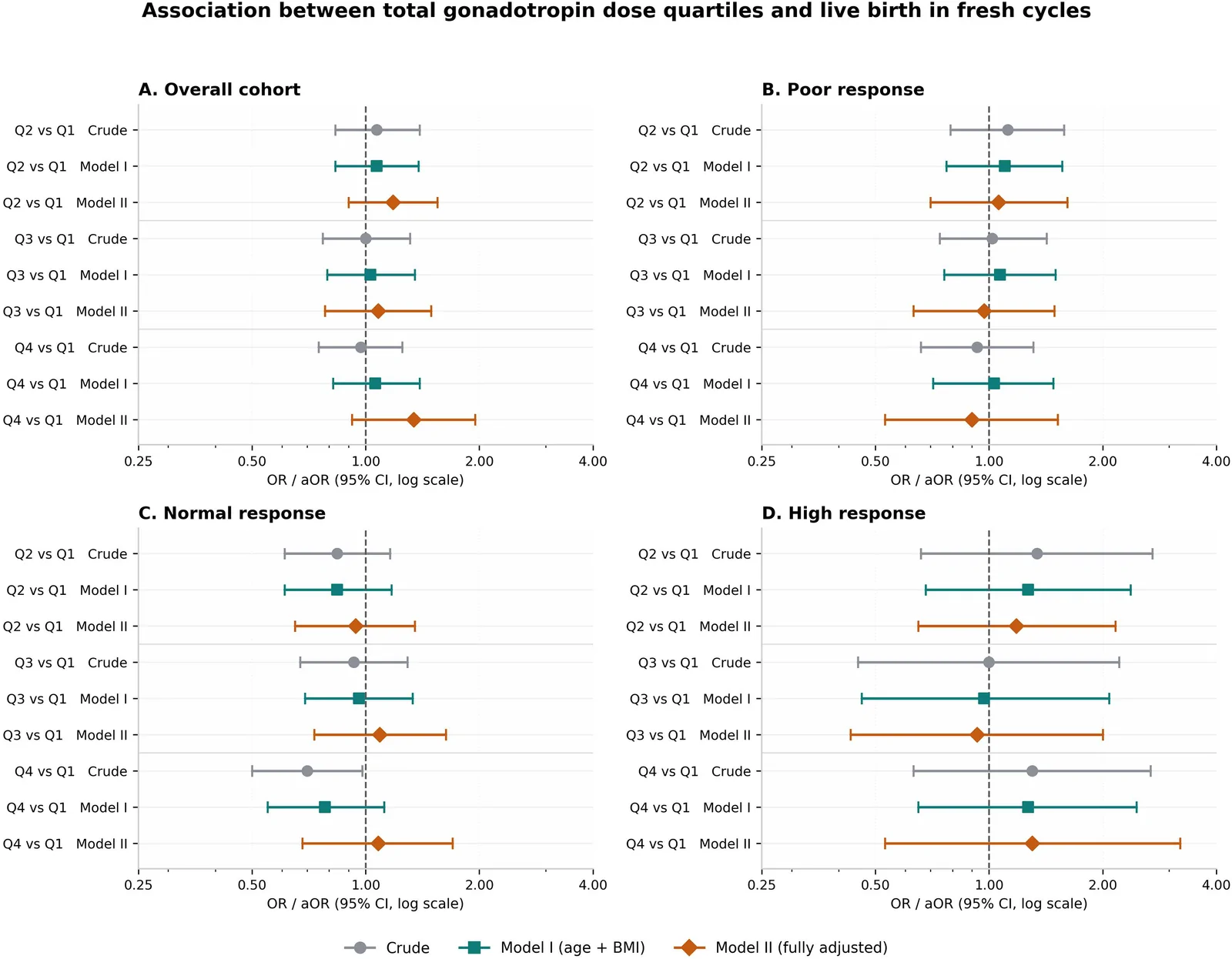
Associations between total Gn dose quartiles and live birth in fresh cycles in the overall cohort **(A)**, poor-response stratum **(B)**, normal-response stratum **(C)**, and high-response stratum **(D)**. Q1 is the reference within each analysis. Model I was adjusted for age and BMI; Model II was fully adjusted and fitted in complete cases (overall n=3,129; poor n=1,133; normal n=1,327; high n=669). Error bars indicate 95% confidence intervals from cluster-robust standard errors.

**Table 3:** Association between whole-cohort total Gn dose quartiles and live birth in fresh cycles.

Contrast (whole cohort)	Live birth rate, %	Crude OR (95% CI)	Model I aOR (95% CI)	Model II aOR (95% CI)	P value (Model II)
Q1 (700–1,650 IU, n=757)	50.6	Reference	Reference	Reference	—
Q2 (1675–2212.5 IU, n=771) vs Q1	52.4	1.07 (0.83–1.39)	1.07 (0.83–1.38)	1.18 (0.90–1.55)	0.231
Q3 (2225–2700 IU, n=836) vs Q1	50.7	1.00 (0.77–1.31)	1.03 (0.79–1.35)	1.08 (0.78–1.49)	0.646
Q4 (2725–8025 IU, n=765) vs Q1	49.8	0.97 (0.75–1.25)	1.06 (0.82–1.39)	1.34 (0.92–1.95)	0.125

Model I was adjusted for female age and BMI. Model II was fully adjusted for the 12 prespecified covariates. Analyses used 3,129 complete-case cycles and cluster-robust standard errors. aOR, adjusted odds ratio; CI, confidence interval; OR, odds ratio.

### 3.5 Poor-response stratum (<10 oocytes retrieved, n=1,346; 1,133 complete cases)

#### 3.5.1 Dose–response relationship between total Gn dose and live birth (RCS)

Patients in the poor-response stratum had the lowest ovarian reserve and the highest Gn consumption (median total Gn dose 2,375 IU; median AMH 2.8 ng/mL). The RCS analysis (**Figure 2B**) showed that the adjusted curve had a shallow inverted-U shape: live birth rose slowly from approximately 62.5% near 1,000 IU to a peak of approximately 64% in the 1,900–2,000 IU range and then declined slowly. The total amplitude was only about 1.5 percentage points, and the 95% confidence band overlapped throughout. The overall dose effect was χ²(3)=1.63 (P = 0.652), and the test for nonlinearity was P = 0.353; neither the overall dose effect nor the nonlinear component was statistically significant. Within the poor-response stratum, no statistically significant dose–response association between total Gn dose and fresh embryo transfer live birth was observed.

#### 3.5.2 Gn dose quartiles and live birth

**Figure 3B**, **Table 4** show live birth rates of 49.8% in Q1 (1,050–1,875 IU), 52.7% in Q2, 50.3% in Q3, and 48.0% in Q4 (3,025–8,025 IU), with no monotonic trend. The crude model and model I gave similar results, and all confidence intervals crossed the null. Two mutually independent analytic strategies—RCS and quartile analysis—reached the same conclusion: within the poor-response stratum, total Gn dose was not significantly associated with live birth.

**Table 4:** Association between within-stratum total Gn dose quartiles and live birth in fresh cycles.

Stratum/contrast	Live birth rate, %	Crude OR (95% CI)	Model I aOR (95% CI)	Model II aOR (95% CI)	P value (Model II)
Poor response (<10 oocytes)					
Q1 (1,050–1,875 IU, n=285)	49.8	Reference	Reference	Reference	—
Q2 (1912–2400 IU, n=298) vs Q1	52.7	1.12 (0.79–1.58)	1.10 (0.77–1.56)	1.06 (0.70–1.61)	0.778
Q3 (2425–3000 IU, n=298) vs Q1	50.3	1.02 (0.74–1.42)	1.07 (0.76–1.50)	0.97 (0.63–1.49)	0.886
Q4 (3025–8025 IU, n=252) vs Q1	48.0	0.93 (0.66–1.31)	1.03 (0.71–1.48)	0.90 (0.53–1.52)	0.698
Normal response (10–15 oocytes)					
Q1 (700–1,650 IU, n=335)	56.7	Reference	Reference	Reference	—
Q2 (1675–2175 IU, n=330) vs Q1	52.4	0.84 (0.61–1.16)	0.84 (0.61–1.17)	0.94 (0.65–1.35)	0.723
Q3 (2200–2662 IU, n=331) vs Q1	55.0	0.93 (0.67–1.29)	0.96 (0.69–1.33)	1.09 (0.73–1.63)	0.657
Q4 (2670–5925 IU, n=331) vs Q1	47.7	0.70 (0.50–0.98)	0.78 (0.55–1.12)	1.08 (0.68–1.70)	0.757
High response (>15 oocytes)					
Q1 (700–1,500 IU, n=173)	44.5	Reference	Reference	Reference	—
Q2 (1525–2025 IU, n=170) vs Q1	51.8	1.34 (0.66–2.71)	1.27 (0.68–2.37)	1.18 (0.65–2.16)	0.584
Q3 (2050–2475 IU, n=189) vs Q1	44.4	1.00 (0.45–2.21)	0.97 (0.46–2.08)	0.93 (0.43–2.00)	0.857
Q4 (2512–6775 IU, n=137) vs Q1	51.1	1.30 (0.63–2.68)	1.27 (0.65–2.46)	1.30 (0.53–3.21)	0.563

Dose quartiles were constructed independently within each response stratum. Complete-case counts were 1,133 poor-response, 1,327 normal-response, and 669 high-response cycles. Models and abbreviations are as in **Table 3**.

#### 3.5.3 Quartile analysis of secondary outcomes in the poor-response stratum

**Table 5**, **Supplementary Table 3** show that none of the 15 quartile comparisons of secondary outcomes reached statistical significance in the unadjusted analysis (minimum P = 0.067), and none was significant after FDR correction for multiple comparisons (minimum adjusted P = 0.675). The point estimate for early miscarriage in Q4 was elevated (aOR=2.61, 95% CI 0.94–7.29, P = 0.067), but this estimate rested on only 69 miscarriage events among 662 clinical pregnancy cycles, with few events, a wide confidence interval, and no dose gradient. Regarding embryo quality indicators, the aORs for the 2PN fertilization rate, D3 good-quality embryo rate, and good-quality blastocyst rate were all close to the null (0.83–1.13); no significant association between Gn dose and embryo quality indicators was observed within this stratum.

**Table 5:** Summary of secondary outcome quartile analyses after false discovery rate control.

Stratum	Outcome	Range of Model II aORs (Q2–Q4 vs Q1)	Minimum FDR q value	Result
Poor response (<10 oocytes)	Clinical pregnancy	0.87–1.00	0.988	Not significant after FDR correction
Poor response (<10 oocytes)	Early miscarriage	0.52–2.61	0.675	Not significant after FDR correction
Poor response (<10 oocytes)	2PN rate	1.01–1.13	0.852	Not significant after FDR correction
Poor response (<10 oocytes)	D3 good-quality embryo rate	0.83–0.98	0.675	Not significant after FDR correction
Poor response (<10 oocytes)	Good-quality blastocyst rate	0.93–0.99	0.988	Not significant after FDR correction
Normal response (10–15 oocytes)	Clinical pregnancy	0.78–1.06	0.452	Not significant after FDR correction
Normal response (10–15 oocytes)	Early miscarriage	0.31–0.58	0.137	Not significant after FDR correction
Normal response (10–15 oocytes)	2PN rate	1.07–1.29	0.137	Not significant after FDR correction
Normal response (10–15 oocytes)	D3 good-quality embryo rate	1.05–1.21	0.202	Not significant after FDR correction
Normal response (10–15 oocytes)	Good-quality blastocyst rate	0.94–1.01	0.872	Not significant after FDR correction
High response (>15 oocytes)	Clinical pregnancy	0.41–0.79	0.302	Not significant after FDR correction
High response (>15 oocytes)	Early miscarriage	0.49–0.93	0.783	Not significant after FDR correction
High response (>15 oocytes)	2PN rate	1.08–1.15	0.783	Not significant after FDR correction
High response (>15 oocytes)	D3 good-quality embryo rate	0.62–1.03	0.302	Not significant after FDR correction
High response (>15 oocytes)	Good-quality blastocyst rate	0.80–0.99	0.783	Not significant after FDR correction

Detailed point estimates and confidence intervals are provided in **Supplementary Table 3**. FDR, false discovery rate; 2PN, two pronuclei.

#### 3.5.4 Sensitivity analyses

Five sensitivity analyses tested the robustness of the stratum’s primary conclusions (**Supplementary Table 4**). (1) Restriction to first oocyte retrieval cycles (n=1,018), to exclude the influence of repeat cycles: the overall dose effect remained nonsignificant (χ²=2.98, P = 0.394). (2) The E-value for the Q4 vs Q1 comparison was 1.46; that is, unmeasured confounding of a strength of only RR ≥1.46 would suffice to fully explain this weak point estimate. (3) Given the marked baseline imbalance between the Q1 and Q4 groups, propensity score overlap weighting was applied (n=537); after weighting, all covariates achieved balance (all SMD <0.1), and the weighted analysis still showed no difference in live birth between Q4 and Q1 (P = 0.719). (4) After exclusion of cycles with BMI ≥28 kg/m² (n=1,070), the dose effect remained nonsignificant (P = 0.628). (5) After multiple imputation of missing covariates with inclusion of all cycles (n=1,346), the aOR for Q4 vs Q1 was 1.20 (95% CI 0.74–1.96, P = 0.460), in the same direction as the complete-case analysis and still nonsignificant. None of the five sensitivity analyses showed a statistically significant association between total Gn dose and fresh-cycle live birth within the poor-response stratum.

#### 3.5.5 Subgroup analysis of poor responders (≤4 vs 5–9 oocytes retrieved)

Within the poor-response stratum, 191 cycles had ≤4 oocytes retrieved and 1,155 cycles had 5–9 oocytes (**Table 6**). Compared with the 5–9 oocyte subgroup, the ≤4 oocyte subgroup had lower ovarian reserve, a higher total Gn dose, and a lower proportion of D5 blastocyst transfer. Within-subgroup dose-quartile analyses showed no statistically significant association between total Gn dose and live birth in either subgroup (**Table 7**; **Supplementary Figure 2**). The within-subgroup RCS analyses likewise showed neither a significant overall dose effect nor significant nonlinearity (P = 0.921 and P = 0.409, respectively) (**Supplementary Figure 1**). When total Gn dose was analyzed as a continuous variable, the model II aOR per 500 IU increase was 0.961 (95% CI 0.748–1.234, P = 0.755) in the ≤4 oocyte subgroup and 0.975 (0.879–1.081, P = 0.625) in the 5–9 oocyte subgroup, and the subgroup × dose interaction was not significant (OR = 0.962, 95% CI 0.790–1.172, P = 0.701) (**Table 7**). The continuous-dose associations with clinical pregnancy or embryo quality indicators were all nonsignificant after FDR correction (**Supplementary Table 6**).

**Table 6:** Characteristics and outcomes of poor-response cycles with ≤4 versus 5–9 oocytes.

Characteristic	≤4 oocytes (n=191)	5–9 oocytes (n=1155)	P value
Female age, years	30.9 ± 3.2	30.7 ± 3.2	0.426
Body mass index, kg/m²	22.2 ± 3.4	22.2 ± 3.2	0.864
Duration of infertility, years	4.0 (2.0–6.0)	3.0 (2.0–5.0)	0.192
Primary infertility, n (%)	79 (41.4)	475 (41.1)	1.000
Basal AMH, ng/mL	2.4 (1.5–4.1)	2.9 (1.9–4.2)	0.007
Total antral follicle count, n	13.0 (9.0–19.5)	15.0 (11.0–21.0)	<0.001
Basal FSH, IU/L	6.0 (5.0–7.1)	5.8 (5.0–6.8)	0.222
Gn starting dose, IU	225.0 (150.0–225.0)	200.0 (150.0–225.0)	0.066
Duration of Gn stimulation, days	11.8 ± 3.0	11.3 ± 2.1	0.048
Total Gn dose, IU	2475.0 (1962.5–3150.0)	2350.0 (1800.0–3000.0)	0.048
Urinary-source Gn use, n (%)	149 (78.0)	864 (74.8)	0.390
Endometrial thickness on trigger day, mm	10.6 (9.3–11.8)	10.6 (9.3–12.0)	0.763
Oocytes retrieved, n	3.0 (3.0–4.0)	7.0 (6.0–8.5)	<0.001
ICSI, n (%)	41 (21.5)	273 (23.6)	0.572
Embryos transferred, n	2.0 (1.0–2.0)	2.0 (1.0–2.0)	0.002
D5 blastocyst transfer, n (%)	5 (2.6)	99 (8.6)	0.007
Clinical pregnancy, n (%)	93 (48.7)	666 (57.7)	0.025
Early miscarriage among clinical pregnancies, n (%)	5 (5.4)	74 (11.1)	0.130
Live birth, n (%)	84 (44.0)	551 (47.7)	0.380

The subgroup analysis was performed to evaluate heterogeneity within the poor-response stratum. Early miscarriage is calculated among clinical pregnancy cycles. AMH, anti-Müllerian hormone; BMI, body mass index; D5, day 5; Gn, gonadotropin; ICSI, intracytoplasmic sperm injection.

**Table 7:** Dose analyses for live birth in fresh cycles within poor-response subgroups.

Analysis/contrast	Live birth, n/N (%)	Crude OR (95% CI)	Model I aOR (95% CI)	Model II aOR (95% CI)	P value
≤4 oocytes					
Q1 (900–1975 IU)	19/41 (46.3)	Reference	Reference	Reference	—
Q2 (2000–2475 IU)	22/41 (53.7)	1.34 (0.56–3.23)	1.35 (0.54–3.38)	1.20 (0.39–3.72)	0.747
Q3 (2550–3150 IU)	18/39 (46.2)	0.99 (0.41–2.42)	1.19 (0.48–2.99)	0.95 (0.29–3.14)	0.934
Q4 (3175–8025 IU)	17/40 (42.5)	0.86 (0.35–2.08)	1.03 (0.40–2.64)	0.87 (0.23–3.23)	0.831
5–9 oocytes					
Q1 (700–1875 IU)	126/251 (50.2)	Reference	Reference	Reference	—
Q2 (1888–2400 IU)	135/255 (52.9)	1.12 (0.77–1.62)	1.11 (0.76–1.62)	1.09 (0.71–1.67)	0.681
Q3 (2425–3000 IU)	129/256 (50.4)	1.01 (0.71–1.43)	0.98 (0.69–1.41)	0.91 (0.59–1.40)	0.673
Q4 (3025–5625 IU)	104/210 (49.5)	0.97 (0.67–1.41)	0.96 (0.65–1.41)	0.84 (0.52–1.37)	0.494
Continuous dose analysis (per 500-IU increment)					
≤4 oocytes	—	—	—	0.961 (0.748–1.234)	0.755
5–9 oocytes	—	—	—	0.975 (0.879–1.081)	0.625
Subgroup × dose interaction	—	—	—	0.962 (0.790–1.172)	0.701

Dose quartiles were constructed separately within the ≤4-oocyte and 5–9-oocyte subgroups. Model II used the same 12 covariates as the primary analysis and cluster-robust standard errors. Complete-case counts were 161 and 972 cycles, respectively. The interaction term evaluates whether the continuous dose association differs between the two subgroups.

### 3.6 Normal-response stratum (10–15 oocytes retrieved, n=1,582; 1,327 complete cases)

#### 3.6.1 Dose–response relationship between total Gn dose and live birth (RCS)

This was the largest subgroup, with a median Gn dose of 2,175 IU and the highest live birth rate of the three strata (50.8%). The RCS analysis (**Figure 2C**) showed that the adjusted dose–response curve in this stratum was approximately horizontal. Over the principal dose range of 1,300–4,000 IU, point estimates of live birth fluctuated within the extremely narrow interval of 62%–63%, with no discernible upward or downward trend. The overall dose effect was χ²(3)=0.21 (P = 0.976), and the test for nonlinearity was P = 0.900; again, neither the overall dose effect nor the nonlinear component was statistically significant. Within the normal-response stratum, no statistically significant dose–response association between total Gn dose and fresh embryo transfer live birth was observed.

#### 3.6.2 Gn dose quartiles and live birth

**Figure 3C**, **Table 4** show live birth rates of 56.7% in Q1, 52.4% in Q2, 55.0% in Q3, and 47.7% in Q4 (2,670–5,925 IU, n=331). With Q1 as reference, the crude OR for Q4 was 0.70 (95% CI 0.50–0.98, P = 0.036). The Q4 group was the subgroup within this stratum with the lowest ovarian reserve, the oldest age, and the highest proportion of urinary Gn use. With sequential adjustment, the ORs for Q2–Q4 vs Q1 progressively approached the null, and in model II all three comparisons had P values exceeding 0.65. After full adjustment, total Gn dose was not significantly associated with fresh-cycle live birth within the normal-response stratum.

#### 3.6.3 Secondary outcomes

**Table 5**, **Supplementary Table 3** show that, of the 15 quartile comparisons of secondary outcomes, 3 reached statistical significance in the analyses unadjusted for multiple comparisons: early miscarriage rate in Q2 (P = 0.017) and 2PN fertilization rate in Q3 and Q4 (aOR=1.21 and 1.29, respectively; P = 0.027 for both comparisons). However, after Benjamini–Hochberg correction, none of the 15 comparisons remained statistically significant (minimum q=0.137). In short, within the normal-response stratum, Gn dose was not significantly associated with embryo quality indicators or early miscarriage.

#### 3.6.4 Sensitivity analyses

Five sensitivity analyses tested the robustness of the null findings in the normal-response stratum (**Supplementary Table 4**). (1) Restriction to first oocyte retrieval cycles (n=1,204), to exclude correlated effects of repeat cycles: the overall dose effect remained nonsignificant (P = 0.943). (2) The E-value for the Q4 vs Q1 point estimate in the primary analysis was 1.36. (3) Propensity score overlap weighting (n=666): given the marked baseline imbalance between the Q1 and Q4 groups, all covariates achieved balance after weighting (SMD <0.1), with weighted live birth rates of 49.2% and 48.7%, respectively (OR = 1.02, 95% CI 0.64–1.62, P = 0.929). (4) Exclusion of cycles with BMI ≥28 kg/m² (n=1,259), to exclude obesity-related effect modification: the overall dose effect remained nonsignificant (P = 1.000). (5) Multiple imputation of missing covariates (n=1,582): aOR=1.23 (95% CI 0.82–1.86, P = 0.317), with a positive point estimate but a wide confidence interval that included the null. None of the five sensitivity analyses showed a statistically significant association (all P>0.3).

### 3.7 High-response stratum (>15 oocytes retrieved, n=783; 669 complete cases)

#### 3.7.1 Dose–response relationship between total Gn dose and live birth (RCS)

The high-response stratum had the best ovarian reserve and the lightest Gn exposure (median Gn dose 2,000 IU) and the smallest sample size of the three strata. The RCS analysis (**Figure 2D**) showed that the adjusted curve had a shallow U shape: live birth declined slowly from approximately 63% near 1,000 IU to approximately 62.7% in the 1,800–2,000 IU range and then rose to approximately 65% at 3,500 IU. The total fluctuation was about 2 percentage points, and the 95% confidence band overlapped throughout. The overall dose effect was χ²(3)=1.36 (P = 0.715), and the test for nonlinearity was P = 0.776; the shallow U shape itself did not depart from the linear null hypothesis. Within the high-response stratum, total Gn dose showed no significant dose–response association with fresh embryo transfer live birth.

#### 3.7.2 Gn dose quartiles and live birth

**Figure 3D**, **Table 4** show that, in the quartile analysis, live birth rates were 44.5% in Q1, 51.8% in Q2, 44.4% in Q3, and 51.1% in Q4, fluctuating irregularly rather than following a monotonic dose gradient. With Q1 as reference, the aORs in the fully adjusted model (model II) were 1.18 (95% CI 0.65–2.16, P = 0.584) for Q2, 0.93 (95% CI 0.43–2.00, P = 0.857) for Q3, and 1.30 (95% CI 0.53–3.21, P = 0.563) for Q4. None of the three comparisons was statistically significant, and the directions of the point estimates were inconsistent. The point estimates were similar across the three models. It should be noted that the confidence intervals for these comparisons were generally wide, reflecting the limited sample size and insufficient estimation precision of this stratum, so that moderate effects in either direction cannot be excluded. In short, within the high-response stratum, total Gn dose was not significantly associated with fresh-cycle live birth, but the precision of this null conclusion is limited by the sample size and should be considered in interpretation.

#### 3.7.3 Secondary outcomes

**Table 5**; **Supplementary Table 3** show that, of the 15 quartile comparisons of secondary outcomes, 2 reached statistical significance in the analyses unadjusted for multiple comparisons: clinical pregnancy rate in Q3 (aOR=0.41, 95% CI 0.19–0.87, P = 0.021) and D3 good-quality embryo rate in Q3 (aOR=0.62, 95% CI 0.39–0.98, P = 0.040). After Benjamini–Hochberg correction for multiple comparisons, none of the 15 comparisons remained statistically significant (minimum q=0.302). No statistical association was observed for the remaining indicators. Within this stratum, total Gn dose was not significantly associated with any secondary outcome, consistent with the null result for the primary outcome.

#### 3.7.4 Sensitivity analyses

Five sensitivity analyses tested the robustness of the null findings in the high-response stratum (**Supplementary Table 4**). (1) Restriction to first oocyte retrieval cycles (n=628), to exclude correlated effects of repeat cycles: the overall dose effect remained nonsignificant (χ²=4.30, P = 0.231). (2) The E-value for the Q4 vs Q1 point estimate in the primary analysis was 1.93; that is, unmeasured confounding of strength RR ≥1.93 would be required to fully explain this point estimate. (3) Propensity score overlap weighting (n=310): given the baseline imbalance between the Q1 and Q4 groups, all covariates achieved balance after weighting (SMD <0.1), with weighted live birth rates of 51.7% and 53.0%, respectively (OR = 0.95, 95% CI 0.40–2.27, P = 0.910). (4) Exclusion of cycles with BMI ≥28 kg/m² (n=625), to exclude obesity-related effect modification: the overall dose effect remained nonsignificant (P = 0.734). (5) Multiple imputation of missing covariates (n=783): aOR=1.37 (95% CI 0.64–2.96, P = 0.416), with the point estimate in the same direction as the complete-case analysis and a wide confidence interval that included the null. The results in this stratum are limited by statistical precision, and small-to-moderate effects cannot be excluded; no sensitivity analysis showed a statistically significant association.

## 4 Discussion

In 3,711 autologous fresh embryo transfer cycles using a single follicular-phase long protocol in women aged ≤35 years, this study systematically evaluated the relationship between total Gn dose and fresh-cycle live birth, and the results consistently showed no significant association. At the overall level, continuous RCS modeling found neither a linear nor a nonlinear association between dose and fresh-cycle live birth. At the stratified level, the within-stratum RCS primary analyses and the sequentially adjusted dose-quartile analyses were null in all three strata—poor (P = 0.652), normal (P = 0.976), and high (P = 0.715) response. For the secondary outcomes, none of the comparisons across the five indicators—2PN fertilization rate, D3 good-quality embryo rate, good-quality blastocyst rate, clinical pregnancy rate, and early miscarriage rate—remained significant after Benjamini–Hochberg correction. For the sensitivity analyses, all five analyses—restriction to first oocyte retrieval cycles, E-values, propensity score overlap weighting, exclusion of obesity, and multiple imputation—reproduced the primary conclusion in every stratum.

### 4.1 Comparison with previous observational evidence

The null results of this study differ from the negative associations reported in previous large-sample studies, the discrepancy arising mainly from differences in dose distribution, population composition, and the depth of confounding control. Analyzing 658,519 fresh cycles from the SART registry, Baker et al. found that live birth declined with increasing total FSH dose within every stratum of oocyte yield (8). However, in that cohort, 32.5% of cycles had doses >4,000 IU and 16.5% had doses ≥5,000 IU, whereas in the present cohort 95% of cycles had doses not exceeding approximately 3,600 IU (P95). Their diagnosis-adjusted models likewise showed that the significant decline was confined to the high-dose segment; that is, the adjusted negative association was concentrated in a high-dose region beyond the principal data range of the present cohort. In addition, that study did not adjust for factors such as AFC and AMH, residual confounding by indication cannot be excluded, and the stratification was relatively coarse (1–5, 6–10 oocytes, etc.). It should also be acknowledged that the present study has a smaller sample size and is restricted to a single stimulation protocol, limiting its ability to extrapolate to extreme doses.

Xu et al. reported a nonlinear inflection point at approximately 1,410 IU in 11,645 non-PCOS fresh cycles (9). However, their fully adjusted logistic model was null (model II OR = 0.96, 95% CI 0.92–1.01), and the inflection point arose mainly from the shape of the GAM smoothing curve; the RCS nonlinearity test in the present study was likewise null (P = 0.701), with no inflection point near 1,410 IU. The differences between the two studies derive from population composition (whether PCOS was excluded) and the choice of dose distribution, and whether that inflection point truly exists remains to be verified. Shen et al., stratifying by AMH, reported that embryo quality and live birth declined with increasing dose (10). Stratification by AMH and stratification by oocyte yield approach the question from different design angles: the former reflects baseline ovarian reserve, within which oocyte yield and dose can still co-vary, whereas the latter fixes the actual level of ovarian response and more directly isolates the association between dose and responsiveness. The two stratification strategies answer different scientific questions, and divergent conclusions are to be expected.

Overall, the disagreement between the present study and previous research genuinely reflects differences in populations, dose ranges, and analytic strategies. The present results should be understood as complementary evidence in the specific context of conventional dose ranges and fixed levels of ovarian response, rather than as a refutation of previous studies. Dose–outcome relationships in larger populations and across wider dose ranges await verification in multicenter, large-sample studies.

### 4.2 Gonadotropin dose and embryo quality: evidence from the PGT era

The hypothesis that high-dose Gn damages embryos has some experimental basis. Supraphysiological FSH levels can disrupt spindle assembly during meiosis I and increase aneuploidy in mouse oocytes (17), and the randomized trial by Baart et al. also suggested that mild stimulation reduces the proportions of embryonic aneuploidy and mosaicism (18). Within the conventional dose range in humans, however, multiple lines of evidence render this hypothesis untenable. First, the principal determinants of oocyte euploidy are age-related cohesin degradation and mitochondrial dysfunction, not stimulation intensity (38). Second, direct evidence from PGT consistently shows that cumulative Gn dose is unrelated to embryonic euploidy: in an analysis of single-embryo-transfer PGS cycles, Barash et al. found that high Gn dose did not affect embryonic euploidy or pregnancy rates (19); Sekhon et al. confirmed in normal responders that, after controlling for age, cumulative Gn dose was unrelated to the risk of embryonic aneuploidy (20); analyzing 12,298 trophectoderm biopsies, Irani et al. showed that neither ovarian stimulation parameters nor the number of oocytes retrieved affected euploidy rates (21); and in a multivariable analysis of 19,246 oocytes, Shuai et al. further confirmed that total Gn dose—whether treated as a continuous or a categorical variable—was unrelated to the euploid blastocyst rate (22).

Two points warrant additional comment. The advantage of mild stimulation in the Baart trial (18) was observed between two groups in which the level of ovarian response was not fixed, and its effect may derive partly from the extremity of the populations and exposures. The present study did not perform embryo biopsy, and embryo quality was assessed entirely with morphological indicators; because morphological appearance does not fully correspond to embryonic ploidy, a genetic-level effect of high-dose Gn on embryos cannot be excluded. The conclusions of this study regarding embryo quality are restricted to morphological endpoints and must be interpreted in conjunction with PGT evidence.

### 4.3 Consistency with randomized trial evidence

The present results agree closely with the available randomized controlled trial evidence at every link of the causal chain. At the level of the starting dose, ESTHER-1 (5), a large non-inferiority RCT of AMH-guided individualized starting doses, showed that individualized dosing improves the predictability of ovarian response and OHSS safety, but the trial was not designed with improved live birth as an objective or an outcome; a dose–response trial stratified by AMH (6) further showed that increasing the starting dose mainly changed the number of oocytes retrieved rather than pregnancy rates; and a prospective study based on an AFC nomogram (7) likewise had optimization of ovarian response as its sole endpoint. At the level of dose-escalation strategies, a randomized comparison of 300 IU vs 225 IU starting doses in predicted normal responders (25) found no improvement in live birth; the study by Out et al. (39) showed that increasing the daily dose cannot compensate for the age-related decline in the number of oocytes that can be obtained; the OPTIMIST trial (26) demonstrated that dose escalation in predicted poor responders neither increased oocyte yield nor improved cumulative live birth, while dose reduction to 100 IU in predicted high responders did not compromise pregnancy outcomes and instead reduced OHSS risk (27). On the basis of this evidence, a Cochrane meta-analysis and a subsequent evidence review (28, 40) both advocated discontinuing the routine use of high-dose FSH in predicted poor responders. At the level of total dose, the present study took the actual total Gn dose consumed as the exposure and confirmed that, at equivalent levels of ovarian response, total dose is not significantly associated with embryo quality or fresh-cycle live birth. Taken together, the evidence at these three levels points to a self-consistent conclusion: Gn dose determines the ceiling on the scale of ovarian response, but under conditions of equal ovarian response, dose per se has no independent improving effect on embryo quality or live birth outcomes.

### 4.4 Physiological basis of the null findings

The null results of this study have a solid basis in follicular physiology. According to the FSH threshold–time window theory (1, 2), the scale of follicular recruitment depends on the length of the time window during which FSH exposure exceeds the threshold, not on the peak serum concentration. With the number of oocytes retrieved fixed (i.e., at equivalent levels of ovarian response), a higher total Gn dose corresponds to only two scenarios. One is a higher daily dose; however, granulosa cell FSH receptors approach saturation at concentrations several times physiological levels, and exposure beyond the saturation threshold produces no additional follicular biological effect. The other is a longer duration of stimulation—that is, extension of the recruitment time window over an established follicle pool, which still recruits the same cohort of follicles. Individual differences in sensitivity caused by FSH receptor gene polymorphisms (41) can explain why the same dose produces markedly different ovarian responses in different patients and form the molecular basis of the between-stratum differences in OSI (34); within a given response stratum, however, individual sensitivity is approximately homogeneous, and dose no longer affects outcomes through the sensitivity pathway. The developmental potential of the oocyte is determined mainly by age-related aging of the meiotic apparatus (38), and FSH exposure within the conventional dose range is insufficient to alter this dominant factor.

### 4.5 Reconciling heterogeneous results in the literature

The mechanistic framework above can coherently explain the contradictory results in the previous literature. The inferior outcomes of high-dose groups in registry studies (8, 42) stem from the directionality of prescribing choices—poorer-prognosis patients receive higher doses—rather than from harm caused by the drug itself. The supraphysiological hormone level–endometrial desynchronization effect suggested by studies comparing fresh and frozen transfer outcomes (23, 24) was not reproduced in the present cohort after the level of ovarian response was controlled (trigger-day endometrial thickness did not differ among the four groups, P = 0.310).

There is a methodological limitation inherent to this study in evaluating that pathway. Per the center’s transfer policy, cycles with trigger-day progesterone ≥1.5 ng/mL had fresh transfer canceled in favor of freeze-all and did not enter the cohort; and the association between elevated trigger-day progesterone and reduced fresh-cycle pregnancy rates has been confirmed by large cohorts and meta-analyses (43, 44). Therefore, this study cannot evaluate any potential effect of high-dose Gn on endometrial receptivity via elevated hormone levels, and the endometrium-related conclusions apply only to the fresh-transfer population with trigger-day progesterone <1.5 ng/mL.

### 4.6 Rationale for stratification by oocyte yield rather than OSI

This study chose the number of oocytes retrieved rather than OSI as the stratification variable for three reasons. First, the research question determines the form of the exposure. Clinical dose decisions are made in IU, whereas OSI can be computed only after oocyte retrieval is complete and therefore cannot serve as a prescribing variable to answer the clinical question of “whether the dose should be increased”; an analysis with OSI as the exposure answers the prognostic question of “whether patients with higher response efficiency have better outcomes,” which differs from the question addressed here. Second, mathematical coupling. OSI is defined as the ratio of the number of oocytes retrieved to the total Gn dose and is highly negatively correlated with dose within each response stratum (Spearman r = −0.75, −0.92, and −0.94, respectively; all P<0.001); analyzing OSI within strata approximates analyzing the reciprocal of dose—the two carry equivalent information, but the former is harder to interpret and artificially introduces nonlinearity. Third, causal structure. OSI is a common descendant of dose (the denominator) and ovarian response (the numerator) and is thus a post-exposure variable; including it in a dose–outcome model amounts to conditioning on a collider, introducing rather than eliminating bias. The standard epidemiological treatment of such ratio-type efficiency variables is to fix their components rather than use the ratio directly; this study followed that principle, stratifying by the number of oocytes retrieved and adjusting for AMH, total antral follicle count, and oocyte yield in model II.

Empirical results support this choice. Fresh-cycle live birth rates across OSI quartiles in this cohort were 46.6%, 49.9%, 49.1%, and 48.6% (P = 0.520), indicating that OSI has no independent predictive value for live birth; when OSI was additionally included in model II, the point estimates for the dose quartile groups were unchanged (e.g., the aOR for Q4 remained 1.06), and the aOR for OSI was 1.00 (95% CI 0.93–1.08, P = 0.997). Once the number of oocytes retrieved and ovarian reserve indicators are included in the model, OSI provides no independent information.

### 4.7 Clinical implications

This study has the following clinical implications. First, at equivalent levels of ovarian response, increasing the total Gn dose is not significantly associated with embryo quality or fresh-cycle live birth, suggesting that within conventional dose ranges, dose escalation solely for the purpose of improving live birth lacks evidential support (25–27). Second, for predicted poor responders with diminished ovarian reserve, follicle pool size is the principal constraint on oocyte yield; empirical dose escalation neither increases oocyte yield (26) nor improves live birth (consistent with the present results in the poor-response stratum) and only increases medication costs and patient burden. Improvement of outcomes in such patients depends on optimization at the level of the stimulation protocol, the value of which awaits further evaluation in prospective studies (28, 40). Third, for patients with adequate ovarian reserve but reduced sensitivity (e.g., obesity or FSH receptor polymorphisms) who genuinely require higher doses to achieve the target response, this study observed no signal within conventional dose ranges that higher doses harm embryo quality or fresh-cycle live birth; clinically, the dose required to achieve the target response should not be withheld out of concern about drug harm, nor should prudent management of high doses be relaxed as a result (45). Fourth, for high responders, dose reduction and mild stimulation do not compromise live birth outcomes and can reduce OHSS risk (27), so dose strategy should be oriented toward OHSS prevention. In addition, cycles with elevated trigger-day progesterone were not included in this study; the choice between fresh and frozen transfer lies beyond the scope of the present evidence and should be individualized on the basis of other evidence. Taken together, the core message of these findings for clinicians is that the principal goal of dose adjustment is to achieve an appropriate ovarian response rather than to pursue any specific total dose, and that the dose should be guided by AMH/AFC and individual patient characteristics, not by presumed effects on embryo quality.

### 4.8 Strengths and limitations

The strengths of this study include: (1) stratification by ovarian response fixed ovarian response efficiency at the design level, constituting a non-parametric mitigation of confounding by indication; (2) restriction to a single stimulation protocol and a single age band markedly reduced population heterogeneity compared with registry studies; (3) multiple morphological endpoints were set along the embryo development chain, with prespecified FDR correction to control multiplicity; (4) five sensitivity analyses cross-validated the primary results—E-values quantified the explanatory threshold for unmeasured confounding (36), and propensity score overlap weighting improved the robustness of estimation in the presence of extreme propensity scores; and (5) repeat cycles from the same patient were handled with cluster-robust standard errors, and the poor-response stratum was further subdivided into ≤4 and 5–9 oocyte subgroups, accommodating heterogeneity within the poor-response population.

This study also has several limitations. (1) The retrospective design cannot preclude residual confounding, and clinicians’ unrecorded prognostic judgments could not be included in the adjustment; although the point estimates were already close to the null, the E-values (1.36–1.93) indicate that unmeasured confounding of moderate strength could theoretically still explain the residual associations (36). (2) Doses in this cohort were concentrated in the conventional range, and the conclusions cannot be extrapolated to extreme exposures >4,000 IU or to other stimulation protocols, especially the antagonist protocol used more commonly in many centers. (3) Only fresh transfer cycles were included; patients undergoing freeze-all (mostly high responders at high OHSS risk) did not enter the cohort, and the conclusions for the high-response stratum are constrained by this selection mechanism. (4) The high-response stratum and the ≤4 oocyte subgroup had limited sample sizes, and some quartile comparisons were underpowered; their null results should be read as precision-limited evidence that small-to-moderate effects cannot yet be excluded, not as proof of zero effect. (5) Embryo biopsy was not performed, and embryo quality assessment was restricted to morphological endpoints; because morphology does not fully correspond to ploidy, a potential genetic-level effect of high-dose Gn cannot be excluded. (6) Per center policy, 317 cycles with trigger-day progesterone ≥1.5 ng/mL (7.7% of the remaining eligible cycles) had fresh transfer canceled and did not enter the cohort; given the strong evidence that elevated progesterone impairs fresh-cycle pregnancy outcomes (43, 44), this study cannot evaluate the effect of high-dose Gn on the endometrium via the hormone-elevation pathway, and the endometrium-related conclusions apply only to the fresh-transfer population with trigger-day progesterone <1.5 ng/mL. (7) Total Gn dose is a composite measure integrating the starting dose, mid-stimulation dose adjustments, and the duration of stimulation, and the present data cannot distinguish these components; mid-stimulation adjustment is routine practice at this center, and the conclusions may not apply to centers that maintain a fixed daily dose and adjust only the duration of stimulation. (8) The number of oocytes retrieved is a post-stimulation variable, and stratification by oocyte yield cannot answer whether higher doses would increase oocyte yield; nor did this study evaluate cumulative live birth across fresh and frozen cycles. (9) The single age band (≤35 years) and this center’s transfer strategy further limit generalizability, the latter differing from the elective single-embryo-transfer and blastocyst-stage strategies of many contemporary centers.

## 5 Conclusion

In autologous fresh embryo transfer cycles in women aged ≤35 years using the follicular-phase long protocol with trigger-day progesterone <1.5 ng/mL, this analysis of 3,711 cycles showed that total Gn dose was not significantly associated with fresh-cycle live birth in the full cohort or within any of the poor-, normal-, and high-response strata; no comparison of morphological embryo quality was statistically significant after FDR correction; and the five sensitivity analyses were consistent. These findings indicate that, within the dose range studied, increasing the Gn dose to improve outcomes lacks evidential support, and moderate dose escalation showed no signal of harm. Dose adjustment should aim to achieve an appropriate ovarian response, determined on the basis of AMH/AFC and individual patient characteristics, rather than presumed effects on embryo quality. Two evidentiary boundaries should be emphasized: the outcomes of this study were limited to morphological embryo quality without embryo biopsy, and cycles with trigger-day progesterone ≥1.5 ng/mL were not included; the associations of Gn dose with embryonic genetic quality and endometrial receptivity await further investigation beyond these boundaries.

## Data Availability

The original contributions presented in the study are included in the article/**Supplementary Material**. Further inquiries can be directed to the corresponding authors.
