# Supplementary material for: Total gonadotropin dose is not associated with live birth or embryo quality in fresh cycles at equivalent levels of ovarian response: a retrospective cohort study stratified by ovarian response
Source: Front Endocrinol (Lausanne). 2026 Oct 7;17:1970297. doi: 10.3389/fendo.2026.1970297 (PMC13683207; doi:10.3389/fendo.2026.1970297)
Supplement: Supplementary file 1 [file SupplementaryFile1.docx]

**Supplementary Tables**

# **Supplementary Tables**

This file contains the full baseline tables and detailed model estimates moved from the main manuscript to improve readability. All estimates are identical to those summarized in the revised manuscript.

# **Supplementary Table S1. Full baseline characteristics of the overall cohort by total Gn dose quartile**

**Table S1. Full baseline characteristics of the overall cohort by total Gn dose quartile.**

| **Variable** | **Q1 (n = 956)** | **Q2 (n = 911)** | **Q3 (n = 964)** | **Q4 (n = 880)** | **P value** |
| --- | --- | --- | --- | --- | --- |
| **Demographic characteristics** |  |  |  |  |  |
| Female age, years | 29.7 ± 3.3 | 30.2 ± 3.2 | 30.6 ± 3.2 | 31.3 ± 3.0 | <0.001 |
| Body mass index, kg/m² | 21.1 ± 2.6 | 21.9 ± 3.0 | 22.5 ± 3.2 | 23.1 ± 3.7 | <0.001 |
| **Infertility history** |  |  |  |  |  |
| Duration of infertility, years | 3.0 (2.0–5.0) | 3.0 (2.0–5.0) | 3.0 (2.0–5.0) | 4.0 (2.0–6.0) | <0.001 |
| Type of infertility, n (%) |  |  |  |  | <0.001 |
| Primary infertility | 459 (48.0) | 415 (45.6) | 378 (39.2) | 369 (41.9) | — |
| Secondary infertility | 496 (51.9) | 495 (54.3) | 586 (60.8) | 510 (58.0) | — |
| History of live birth, n (%) | 214 (22.4) | 212 (23.3) | 295 (30.6) | 267 (30.3) | <0.001 |
| History of miscarriage, n (%) | 240 (25.1) | 221 (24.3) | 276 (28.6) | 203 (23.1) | 0.037 |
| **Ovarian reserve and basal hormones** |  |  |  |  |  |
| Basal AMH, ng/mL | 5.0 (3.7–6.7) | 3.6 (2.8–5.0) | 3.0 (2.2–4.2) | 2.4 (1.7–3.4) | <0.001 |
| Total antral follicle count, n | 22.0 (16.8–25.0) | 18.0 (14.0–23.0) | 16.0 (12.0–20.0) | 13.0 (10.0–18.0) | <0.001 |
| Basal FSH, IU/L | 5.2 (4.5–6.0) | 5.5 (4.8–6.5) | 5.7 (4.9–6.5) | 6.0 (5.1–6.9) | <0.001 |
| Basal LH, IU/L | 3.8 (2.8–5.7) | 3.3 (2.5–4.7) | 3.0 (2.2–4.2) | 2.9 (2.2–4.1) | <0.001 |
| Basal E2, pg/mL | 29.0 (22.0–41.0) | 29.0 (22.0–41.0) | 29.0 (22.0–40.0) | 30.0 (22.0–40.0) | 0.895 |
| Basal PRL, ng/mL | 16.0 (11.2–22.4) | 15.7 (11.0–22.2) | 14.4 (10.7–21.6) | 14.2 (10.3–21.1) | 0.002 |
| Basal T, ng/dL | 0.3 (0.3–0.4) | 0.3 (0.2–0.4) | 0.3 (0.2–0.4) | 0.3 (0.2–0.4) | 0.739 |
| Basal P, ng/mL | 0.3 (0.2–0.4) | 0.3 (0.2–0.4) | 0.2 (0.2–0.4) | 0.2 (0.2–0.4) | 0.625 |
| **Gn medication and stimulation course** |  |  |  |  |  |
| Gn starting dose, IU | 150.0 (112.5–150.0) | 150.0 (150.0–200.0) | 225.0 (150.0–225.0) | 225.0 (225.0–300.0) | <0.001 |
| Total Gn duration, days | 10.1 ± 1.4 | 10.8 ± 1.6 | 11.3 ± 1.6 | 12.8 ± 2.4 | <0.001 |
| Total Gn dose, IU | 1425.0 (1246.9–1550.0) | 1987.5 (1800.0–2062.5) | 2475.0 (2325.0–2600.0) | 3225.0 (2925.0–3600.0) | <0.001 |
| Total HMG dose, IU | 225.0 (112.5–375.0) | 375.0 (225.0–450.0) | 375.0 (225.0–525.0) | 525.0 (412.5–750.0) | <0.001 |
| Urinary-source Gn use, n (%) | 456 (47.7) | 599 (65.8) | 744 (77.2) | 828 (94.1) | <0.001 |
| Gn medication type, n (%) |  |  |  |  | <0.001 |
| Exclusively domestic drugs | 356 (37.2) | 441 (48.4) | 625 (64.8) | 625 (71.0) | — |
| Exclusively imported drugs | 321 (33.6) | 181 (19.9) | 77 (8.0) | 15 (1.7) | — |
| Mixed use | 279 (29.2) | 289 (31.7) | 262 (27.2) | 240 (27.3) | — |
| FSH on Gn start day, IU/L | 2.8 (2.0–3.7) | 2.7 (1.8–3.8) | 2.6 (1.9–3.6) | 2.3 (1.7–3.2) | <0.001 |
| LH on Gn start day, IU/L | 0.5 (0.4–0.7) | 0.5 (0.3–0.6) | 0.4 (0.3–0.6) | 0.4 (0.3–0.5) | <0.001 |
| E2 on Gn start day, pg/mL | 10.0 (10.0–16.0) | 10.0 (10.0–13.0) | 10.0 (10.0–12.0) | 10.0 (10.0–13.0) | <0.001 |
| **Trigger-day indicators and oocyte yield** |  |  |  |  |  |
| E2 on trigger day, pg/mL | 2634.5 (1902.8–3430.0) | 2337.0 (1657.8–3146.0) | 2250.0 (1658.5–3048.2) | 2001.5 (1402.0–2781.2) | <0.001 |
| LH on trigger day, IU/L | 0.8 (0.6–1.0) | 0.7 (0.6–1.0) | 0.7 (0.6–1.0) | 0.7 (0.6–0.9) | 0.410 |
| Progesterone on trigger day, ng/mL | 0.5 (0.3–0.7) | 0.6 (0.4–0.8) | 0.6 (0.4–0.8) | 0.6 (0.4–0.8) | <0.001 |
| Follicle count on trigger day, n | 11.7 ± 3.8 | 10.2 ± 3.6 | 9.7 ± 3.6 | 8.7 ± 3.5 | <0.001 |
| Endometrial thickness on trigger day, mm | 10.5 (9.2–11.9) | 10.7 (9.3–12.0) | 10.6 (9.4–12.0) | 10.8 (9.3–12.0) | 0.310 |
| Follicles punctured, n | 16.0 ± 5.2 | 13.9 ± 5.0 | 13.5 ± 4.8 | 11.9 ± 4.6 | <0.001 |
| Oocytes retrieved, n | 12.7 ± 4.6 | 11.7 ± 4.6 | 11.6 ± 4.6 | 10.0 ± 4.5 | <0.001 |
| Mature oocytes, n | 11.3 ± 4.3 | 10.6 ± 4.4 | 10.6 ± 4.5 | 9.0 ± 4.2 | <0.001 |
| Normally fertilized zygotes, n | 7.3 ± 3.7 | 7.0 ± 3.6 | 7.2 ± 3.9 | 6.0 ± 3.4 | <0.001 |
| OSI | 9.1 (6.7–11.8) | 5.9 (4.3–7.6) | 4.7 (3.3–6.0) | 2.8 (2.0–3.9) | <0.001 |
| **Insemination method and transfer characteristics** |  |  |  |  |  |
| Insemination method, n (%) |  |  |  |  | 0.716 |
| IVF | 722 (75.5) | 676 (74.2) | 711 (73.8) | 666 (75.7) | — |
| ICSI | 234 (24.5) | 235 (25.8) | 253 (26.2) | 214 (24.3) | — |
| Embryos transferred, n | 1.7 ± 0.5 | 1.6 ± 0.5 | 1.6 ± 0.5 | 1.7 ± 0.5 | 0.022 |
| Number of embryos transferred, n (%) |  |  |  |  | 0.022 |
| One embryo transferred | 322 (33.7) | 364 (40.0) | 353 (36.6) | 302 (34.3) | — |
| Two embryos transferred | 634 (66.3) | 547 (60.0) | 611 (63.4) | 578 (65.7) | — |
| Type of embryos transferred, n (%) |  |  |  |  | <0.001 |
| D3 cleavage-stage embryo transfer | 710 (74.3) | 712 (78.2) | 751 (77.9) | 735 (83.5) | — |
| D5 blastocyst transfer | 246 (25.7) | 199 (21.8) | 213 (22.1) | 145 (16.5) | — |

Q1–Q4 are whole-cohort quartiles of total Gn dose. Continuous variables are mean ± SD or median (IQR), as appropriate; categorical variables are n (%). AMH, anti-Müllerian hormone; E2, estradiol; FSH, follicle-stimulating hormone; Gn, gonadotropin; HMG, human menopausal gonadotropin; ICSI, intracytoplasmic sperm injection; LH, luteinizing hormone; OSI, ovarian sensitivity index; PRL, prolactin; T, testosterone.

# **Supplementary Table S2. Full characteristics and outcomes across ovarian response strata**

**Table S2. Full characteristics and outcomes of the three ovarian response strata.**

| **Characteristic** | **Poor response (<10 oocytes, n = 1,346)** | **Normal response (10–15 oocytes, n = 1,582)** | **High response (>15 oocytes, n = 783)** | **P value** |
| --- | --- | --- | --- | --- |
| **Demographic and ovarian reserve characteristics** |  |  |  |  |
| Female age, years | 30.7 ± 3.2 | 30.5 ± 3.2 | 29.9 ± 3.4 | <0.001 |
| Body mass index, kg/m² | 22.2 ± 3.2 | 22.1 ± 3.2 | 22.0 ± 3.3 | 0.673 |
| Basal AMH, ng/mL | 2.8 (1.8–4.2) | 3.6 (2.5–5.0) | 4.3 (3.1–5.7) | <0.001 |
| Total antral follicle count, n | 15.0 (11.0–21.0) | 18.0 (14.0–23.0) | 19.0 (15.0–24.0) | <0.001 |
| Type of infertility, n (%) |  |  |  | 0.058 |
| Primary infertility | 554 (41.2) | 710 (44.9) | 357 (45.6) | — |
| Secondary infertility | 792 (58.8) | 869 (54.9) | 426 (54.4) | — |
| **Gn medication and ovarian response** |  |  |  |  |
| Gn starting dose, IU | 200.0 (150.0–225.0) | 150.0 (150.0–225.0) | 150.0 (150.0–225.0) | <0.001 |
| Total Gn duration, days | 11.4 ± 2.2 | 11.2 ± 1.9 | 11.1 ± 1.8 | 0.005 |
| Total Gn dose, IU | 2375.0 (1856.2–3000.0) | 2175.0 (1650.0–2625.0) | 2000.0 (1500.0–2412.5) | <0.001 |
| Gn medication type, n (%) |  |  |  | <0.001 |
| Exclusively domestic drugs | 785 (58.3) | 878 (55.5) | 384 (49.0) | — |
| Exclusively imported drugs | 173 (12.9) | 268 (16.9) | 153 (19.5) | — |
| Mixed use | 388 (28.8) | 436 (27.6) | 246 (31.4) | — |
| Oocytes retrieved, n | 6.7 ± 2.0 | 12.3 ± 1.6 | 18.3 ± 2.2 | <0.001 |
| OSI | 2.8 (2.1–4.0) | 5.7 (4.5–7.4) | 9.4 (7.4–12.0) | <0.001 |
| **Fertilization and transfer characteristics** |  |  |  |  |
| Insemination method, n (%) |  |  |  | 0.128 |
| IVF | 1032 (76.7) | 1169 (73.9) | 574 (73.3) | — |
| ICSI | 314 (23.3) | 413 (26.1) | 209 (26.7) | — |
| Number of embryos transferred, n (%) |  |  |  | <0.001 |
| One embryo transferred | 436 (32.4) | 544 (34.4) | 361 (46.1) | — |
| Two embryos transferred | 910 (67.6) | 1038 (65.6) | 422 (53.9) | — |
| Type of embryos transferred, n (%) |  |  |  | <0.001 |
| D3 cleavage-stage embryo transfer | 1241 (92.2) | 1263 (79.8) | 404 (51.6) | — |
| D5 blastocyst transfer | 105 (7.8) | 319 (20.2) | 379 (48.4) | — |
| **Pregnancy outcomes** |  |  |  |  |
| Clinical pregnancy rate, n (%) | 759 (56.4) | 933 (59.0) | 479 (61.2) | 0.085 |
| Early miscarriage rate, n (%)* | 79 (10.4) | 71 (7.6) | 57 (11.9) | 0.020 |
| Live birth in fresh cycles, n (%) | 635 (47.2) | 804 (50.8) | 362 (46.2) | 0.051 |
| **Embryology laboratory indicators†** |  |  |  |  |
| 2PN fertilization rate, % | 66.4 | 66.6 | 64.8 | — |
| D3 good-quality embryo rate, % | 53.5 | 52.4 | 50.3 | — |
| Blastocyst formation rate, % | 69.7 | 70.8 | 72.4 | — |
| Good-quality blastocyst rate, % | 26.5 | 26.8 | 27.6 | — |

*Early miscarriage is calculated among clinical pregnancy cycles. Laboratory indicators are pooled cycle-level rates. 2PN, two pronuclei; D3, day 3; D5, day 5; IVF, in vitro fertilization; other abbreviations as in Table S1.

# **Supplementary Table S3. Detailed secondary-outcome dose-quartile analyses**

**Table S3. Detailed secondary outcome analyses by total Gn dose quartile within each response stratum.**

| **Stratum / outcome** | **Contrast** | **N** | **aOR (95% CI)** | **P value** | **FDR q value** |
| --- | --- | --- | --- | --- | --- |
| **Poor response (<10 oocytes)** |  |  |  |  |  |
| Clinical pregnancy | Q2 vs Q1 | 1133 | 0.87 (0.57–1.34) | 0.538 | 0.988 |
| Clinical pregnancy | Q3 vs Q1 | 1133 | 1.00 (0.65–1.54) | 0.988 | 0.988 |
| Clinical pregnancy | Q4 vs Q1 | 1133 | 0.94 (0.56–1.59) | 0.827 | 0.988 |
| Early miscarriage* | Q2 vs Q1 | 662 | 0.52 (0.20–1.34) | 0.174 | 0.675 |
| Early miscarriage* | Q3 vs Q1 | 662 | 2.18 (0.80–5.93) | 0.128 | 0.675 |
| Early miscarriage* | Q4 vs Q1 | 662 | 2.61 (0.94–7.29) | 0.067 | 0.675 |
| 2PN rate† | Q2 vs Q1 | 582 | 1.04 (0.85–1.27) | 0.719 | 0.988 |
| 2PN rate† | Q3 vs Q1 | 583 | 1.01 (0.82–1.26) | 0.915 | 0.988 |
| 2PN rate† | Q4 vs Q1 | 537 | 1.13 (0.88–1.44) | 0.341 | 0.852 |
| D3 good-quality embryo rate† | Q2 vs Q1 | 576 | 0.86 (0.67–1.10) | 0.227 | 0.682 |
| D3 good-quality embryo rate† | Q3 vs Q1 | 573 | 0.98 (0.78–1.22) | 0.849 | 0.988 |
| D3 good-quality embryo rate† | Q4 vs Q1 | 531 | 0.83 (0.64–1.09) | 0.180 | 0.675 |
| Good-quality blastocyst rate† | Q2 vs Q1 | 464 | 0.97 (0.69–1.34) | 0.834 | 0.988 |
| Good-quality blastocyst rate† | Q3 vs Q1 | 463 | 0.99 (0.67–1.47) | 0.974 | 0.988 |
| Good-quality blastocyst rate† | Q4 vs Q1 | 423 | 0.93 (0.56–1.53) | 0.772 | 0.988 |
| **Normal response (10–15 oocytes)** |  |  |  |  |  |
| Clinical pregnancy | Q2 vs Q1 | 1327 | 0.78 (0.53–1.13) | 0.183 | 0.452 |
| Clinical pregnancy | Q3 vs Q1 | 1327 | 1.06 (0.70–1.60) | 0.788 | 0.908 |
| Clinical pregnancy | Q4 vs Q1 | 1327 | 0.87 (0.55–1.40) | 0.577 | 0.866 |
| Early miscarriage* | Q2 vs Q1 | 785 | 0.31 (0.12–0.81) | 0.017 | 0.137 |
| Early miscarriage* | Q3 vs Q1 | 785 | 0.58 (0.24–1.37) | 0.211 | 0.452 |
| Early miscarriage* | Q4 vs Q1 | 785 | 0.42 (0.17–1.08) | 0.072 | 0.217 |
| 2PN rate† | Q2 vs Q1 | 665 | 1.07 (0.91–1.25) | 0.397 | 0.661 |
| 2PN rate† | Q3 vs Q1 | 666 | 1.21 (1.02–1.44) | 0.027 | 0.137 |
| 2PN rate† | Q4 vs Q1 | 663 | 1.29 (1.03–1.62) | 0.027 | 0.137 |
| D3 good-quality embryo rate† | Q2 vs Q1 | 664 | 1.08 (0.91–1.28) | 0.391 | 0.661 |
| D3 good-quality embryo rate† | Q3 vs Q1 | 665 | 1.21 (1.00–1.48) | 0.054 | 0.202 |
| D3 good-quality embryo rate† | Q4 vs Q1 | 662 | 1.05 (0.83–1.33) | 0.698 | 0.872 |
| Good-quality blastocyst rate† | Q2 vs Q1 | 634 | 1.01 (0.81–1.27) | 0.908 | 0.908 |
| Good-quality blastocyst rate† | Q3 vs Q1 | 641 | 0.94 (0.74–1.21) | 0.649 | 0.872 |
| Good-quality blastocyst rate† | Q4 vs Q1 | 638 | 0.98 (0.72–1.33) | 0.872 | 0.908 |
| **High response (>15 oocytes)** |  |  |  |  |  |
| Clinical pregnancy | Q2 vs Q1 | 669 | 0.79 (0.45–1.38) | 0.401 | 0.783 |
| Clinical pregnancy | Q3 vs Q1 | 669 | 0.41 (0.19–0.87) | 0.021 | 0.302 |
| Clinical pregnancy | Q4 vs Q1 | 669 | 0.50 (0.21–1.20) | 0.121 | 0.605 |
| Early miscarriage* | Q2 vs Q1 | 405 | 0.49 (0.11–2.14) | 0.342 | 0.783 |
| Early miscarriage* | Q3 vs Q1 | 405 | 0.93 (0.30–2.90) | 0.905 | 0.944 |
| Early miscarriage* | Q4 vs Q1 | 405 | 0.85 (0.22–3.22) | 0.807 | 0.944 |
| 2PN rate† | Q2 vs Q1 | 343 | 1.08 (0.86–1.37) | 0.500 | 0.783 |
| 2PN rate† | Q3 vs Q1 | 362 | 1.15 (0.88–1.51) | 0.290 | 0.783 |
| 2PN rate† | Q4 vs Q1 | 310 | 1.11 (0.81–1.52) | 0.522 | 0.783 |
| D3 good-quality embryo rate† | Q2 vs Q1 | 342 | 1.03 (0.80–1.32) | 0.832 | 0.944 |
| D3 good-quality embryo rate† | Q3 vs Q1 | 362 | 0.62 (0.39–0.98) | 0.040 | 0.302 |
| D3 good-quality embryo rate† | Q4 vs Q1 | 310 | 0.97 (0.68–1.40) | 0.882 | 0.944 |
| Good-quality blastocyst rate† | Q2 vs Q1 | 333 | 0.99 (0.74–1.32) | 0.944 | 0.944 |
| Good-quality blastocyst rate† | Q3 vs Q1 | 336 | 0.80 (0.57–1.13) | 0.210 | 0.783 |
| Good-quality blastocyst rate† | Q4 vs Q1 | 303 | 0.85 (0.56–1.30) | 0.463 | 0.783 |

Q1 is the reference within each stratum. Clinical pregnancy and early miscarriage were analyzed by logistic regression; rate outcomes were analyzed by binomial generalized linear models with appropriate denominators. FDR correction was applied within each stratum across 15 comparisons.

# **Supplementary Table S4. Itemized sensitivity analyses**

**Table S4. Itemized sensitivity analyses within each ovarian response stratum.**

| **Stratum / sensitivity analysis** | **Analysis set** | **Effect estimate** | **P value** | **Conclusion** |
| --- | --- | --- | --- | --- |
| **Poor response (<10 oocytes)** |  |  |  |  |
| ① First oocyte retrieval cycles (RCS overall dose effect) | N=1018 | Wald χ²(3)=2.98 | 0.394 | Consistent with main analysis |
| ② E-value (Q4 vs Q1 point estimate) | — | aOR=0.90 → E-value=1.46 | — | Explanation by residual confounding unlikely |
| ③ Propensity score overlap weighting (Q1 vs Q4, ATO) | N=537 | Weighted live birth rate 46.7% vs 49.2%; OR=0.91 (0.53–1.55) | 0.719 | SMD 1.36→0.000; consistent with main analysis |
| ④ Exclusion of BMI ≥28 kg/m² (RCS overall dose effect) | N=1070 | Wald χ²(3)=1.74 | 0.628 | Consistent with main analysis |
| ⑤ Multiple imputation (5 datasets, Q4 vs Q1) | N=1346 | aOR=1.20 (0.74–1.96) | 0.460 | Consistent with main analysis |
| **Normal response (10–15 oocytes)** |  |  |  |  |
| ① First oocyte retrieval cycles (RCS overall dose effect) | N=1204 | Wald χ²(3)=0.39 | 0.943 | Consistent with main analysis |
| ② E-value (Q4 vs Q1 point estimate) | — | aOR=1.08 → E-value=1.36 | — | Explanation by residual confounding unlikely |
| ③ Propensity score overlap weighting (Q1 vs Q4, ATO) | N=666 | Weighted live birth rate 49.2% vs 48.7%; OR=1.02 (0.64–1.62) | 0.929 | SMD 1.30→0.000; consistent with main analysis |
| ④ Exclusion of BMI ≥28 kg/m² (RCS overall dose effect) | N=1259 | Wald χ²(3)=0.00 | 1.000 | Consistent with main analysis |
| ⑤ Multiple imputation (5 datasets, Q4 vs Q1) | N=1582 | aOR=1.23 (0.82–1.86) | 0.317 | Consistent with main analysis |
| **High response (>15 oocytes)** |  |  |  |  |
| ① First oocyte retrieval cycles (RCS overall dose effect) | N=628 | Wald χ²(3)=4.30 | 0.231 | Consistent with main analysis |
| ② E-value (Q4 vs Q1 point estimate) | — | aOR=1.30 → E-value=1.93 | — | Explanation by residual confounding unlikely |
| ③ Propensity score overlap weighting (Q1 vs Q4, ATO) | N=310 | Weighted live birth rate 51.7% vs 53.0%; OR=0.95 (0.40–2.27) | 0.910 | SMD 1.05→0.000; consistent with main analysis |
| ④ Exclusion of BMI ≥28 kg/m² (RCS overall dose effect) | N=625 | Wald χ²(3)=1.28 | 0.734 | Consistent with main analysis |
| ⑤ Multiple imputation (5 datasets, Q4 vs Q1) | N=783 | aOR=1.37 (0.64–2.96) | 0.416 | Consistent with main analysis |

RCS, restricted cubic spline; PS, propensity score; ATO, average treatment effect in the overlap population; BMI, body mass index; SMD, standardized mean difference.

# **Supplementary Table S5. Full baseline table for poor-response subgroups**

**Table S5. Baseline characteristics and outcomes of poor-response cycles with ≤4 versus 5–9 oocytes.**

| **Characteristic** | **≤4 oocytes (n=191)** | **5–9 oocytes (n=1155)** | **P value** |
| --- | --- | --- | --- |
| Female age, years | 30.9 ± 3.2 | 30.7 ± 3.2 | 0.426 |
| Body mass index, kg/m² | 22.2 ± 3.4 | 22.2 ± 3.2 | 0.864 |
| Duration of infertility, years | 4.0 (2.0–6.0) | 3.0 (2.0–5.0) | 0.192 |
| Primary infertility, n (%) | 79 (41.4) | 475 (41.1) | 1.000 |
| History of live birth, n (%) | 55 (28.8) | 293 (25.4) | 0.361 |
| History of miscarriage, n (%) | 52 (27.2) | 329 (28.5) | 0.786 |
| Basal AMH, ng/mL | 2.4 (1.5–4.1) | 2.9 (1.9–4.2) | 0.007 |
| Total antral follicle count, n | 13.0 (9.0–19.5) | 15.0 (11.0–21.0) | <0.001 |
| Basal FSH, IU/L | 6.0 (5.0–7.1) | 5.8 (5.0–6.8) | 0.222 |
| Gn starting dose, IU | 225.0 (150.0–225.0) | 200.0 (150.0–225.0) | 0.066 |
| Duration of Gn stimulation, days | 11.8 ± 3.0 | 11.3 ± 2.1 | 0.048 |
| Total Gn dose, IU | 2475.0 (1962.5–3150.0) | 2350.0 (1800.0–3000.0) | 0.048 |
| Urinary-source Gn use, n (%) | 149 (78.0) | 864 (74.8) | 0.390 |
| Endometrial thickness on trigger day, mm | 10.6 (9.3–11.8) | 10.6 (9.3–12.0) | 0.763 |
| Oocytes retrieved, n | 3.0 (3.0–4.0) | 7.0 (6.0–8.5) | <0.001 |
| ICSI, n (%) | 41 (21.5) | 273 (23.6) | 0.572 |
| Embryos transferred, n | 2.0 (1.0–2.0) | 2.0 (1.0–2.0) | 0.002 |
| D5 blastocyst transfer, n (%) | 5 (2.6) | 99 (8.6) | 0.007 |
| Clinical pregnancy, n (%) | 93 (48.7) | 666 (57.7) | 0.025 |
| Early miscarriage among clinical pregnancies, n (%) | 5 (5.4) | 74 (11.1) | 0.130 |
| Live birth, n (%) | 84 (44.0) | 551 (47.7) | 0.380 |

Early miscarriage is calculated among clinical pregnancy cycles. AMH, anti-Müllerian hormone; AFC, antral follicle count; BMI, body mass index; D5, day 5; Gn, gonadotropin; ICSI, intracytoplasmic sperm injection.

# **Supplementary Table S6. Continuous-dose analyses in poor-response subgroups**

**Table S6. Fully adjusted associations per 500-IU increase in total Gn dose with primary and secondary outcomes in poor-response subgroups.**

| **Outcome** | **Cycles with data** | **Outcome denominator** | **Model II aOR (95% CI)** | **P value** | **FDR q within subgroup** | **FDR q across subgroup outcomes** |
| --- | --- | --- | --- | --- | --- | --- |
| **≤4 oocytes** |  |  |  |  |  |  |
| Live birth | 161 |  | 0.961 (0.748–1.234) | 0.755 | 0.755 | 0.906 |
| Clinical pregnancy | 161 |  | 0.926 (0.704–1.219) | 0.585 | 0.702 | 0.905 |
| 2PN fertilization rate | 160 | 482 | 0.944 (0.819–1.088) | 0.427 | 0.702 | 0.905 |
| D3 good-quality embryo rate | 154 | 350 | 0.944 (0.796–1.120) | 0.510 | 0.702 | 0.905 |
| Blastocyst formation rate | 65 | 93 | 0.485 (0.216–1.090) | 0.080 | 0.479 | 0.905 |
| Good-quality blastocyst rate | 43 | 58 | 0.700 (0.312–1.569) | 0.386 | 0.702 | 0.905 |
| **5–9 oocytes** |  |  |  |  |  |  |
| Live birth | 972 |  | 0.975 (0.879–1.081) | 0.625 | 0.990 | 0.905 |
| Clinical pregnancy | 972 |  | 1.001 (0.899–1.114) | 0.990 | 0.990 | 0.990 |
| 2PN fertilization rate | 972 | 6604 | 1.014 (0.963–1.069) | 0.592 | 0.990 | 0.905 |
| D3 good-quality embryo rate | 962 | 4392 | 0.975 (0.919–1.034) | 0.399 | 0.990 | 0.905 |
| Blastocyst formation rate | 825 | 2914 | 0.984 (0.914–1.060) | 0.679 | 0.990 | 0.905 |
| Good-quality blastocyst rate | 739 | 2043 | 1.004 (0.910–1.107) | 0.944 | 0.990 | 0.990 |

For live birth and clinical pregnancy, the denominator is the number of cycles; for early miscarriage, clinical pregnancies; and for laboratory rates, the corresponding oocyte/embryo denominator. Early miscarriage was not modeled in the ≤4-oocyte subgroup because only five events occurred.

# Supplementary Figures

## Supplementary Figure S1. Dose–response curves within poor-response subgroups (RCS)

Supplementary Figure S1. Conditional adjusted dose–response relationships between total Gn dose and live birth in fresh cycles within the poor-response subgroups: ≤4 oocytes (A) and 5–9 oocytes (B). Curves are conditional predicted probabilities of live birth at the reference covariate profile (continuous covariates at subgroup means; categorical covariates at the most frequent category) from fully adjusted restricted cubic spline models with four knots at the 5th, 35th, 65th, and 95th percentiles, fitted in complete cases (n=161 and n=972, respectively) with cluster-robust standard errors. Shaded areas are 95% confidence bands; histograms show the dose distributions.

## Supplementary Figure S2. Dose-quartile forest plots within poor-response subgroups

Supplementary Figure S2. Associations between within-subgroup total Gn dose quartiles and live birth in fresh cycles in poor-response cycles with ≤4 oocytes (A) and 5–9 oocytes (B). Q1 is the reference within each subgroup. Model I was adjusted for age and BMI; Model II was fully adjusted for the 12 prespecified covariates. The bottom row of each panel shows the Model II estimate per 500-IU increment. Error bars indicate 95% confidence intervals from cluster-robust standard errors.
